# Porphyrins as Chiroptical Conformational Probes for Biomolecules

**DOI:** 10.3390/molecules30071512

**Published:** 2025-03-28

**Authors:** Gabriele Travagliante, Massimiliano Gaeta, Roberto Purrello, Alessandro D’Urso

**Affiliations:** Dipartimento di Scienze Chimiche, Università degli Studi di Catania, Viale Andrea Doria, 6, 95125 Catania, Italy; travagliantegab@gmail.com (G.T.); massimiliano.gaeta@unict.it (M.G.); rpurrello@unict.it (R.P.)

**Keywords:** porphyrins, circular dichroism, chiroptical probes, nucleic acids, DNA polymorphism, RNA structures, biomolecules

## Abstract

Porphyrins are highly conjugated macrocyclic compounds that possess exceptional photophysical and chemical properties, progressively establishing themselves as versatile tools in the structural investigation of biomolecules. This review explores their role as chiroptical conformational probes, focusing on their interactions with DNA and RNA. The planar electron rich structure of porphyrin macrocycle that promote π–π interactions, their easy functionalization at the meso positions, and their capacity to coordinate metal ions enable their use in probing nucleic acid structures with high sensitivity. Emphasis is placed on their induced circular dichroism (ICD) signals in the Soret region, which provide precise diagnostic insights into binding mechanisms and molecular interactions. The review examines the interactions of porphyrins with various DNA structures, including B-, Z-, and A-DNA, single-stranded DNA, and G-quadruplex DNA, as well as less common structures like I-motif and E-motif DNA. The last part highlights recent advancements in the use of porphyrins to probe RNA structures, emphasizing binding behaviors and chiroptical signals observed with RNA G-quadruplexes, as well as the challenges in interpreting ICD signals with other RNA motifs due to their inherent structural complexity.

## 1. Introduction

Porphyrins, deriving their name from the Greek word πορφύρα (porphura, meaning purple), represent a fascinating class of macrocyclic compounds fundamental in numerous biological processes, including oxygen transport, photosynthesis, and catalysis [1].

Often referred to as the pigments of life [2], these compounds are exemplified by chlorophylls and hemes. Chlorophylls play essential roles in photosynthesis as light-harvesting antennas and charge separation facilitators, while hemes are critical for biocatalysis and oxygen transport in the bloodstream [3].

**Figure 1:**
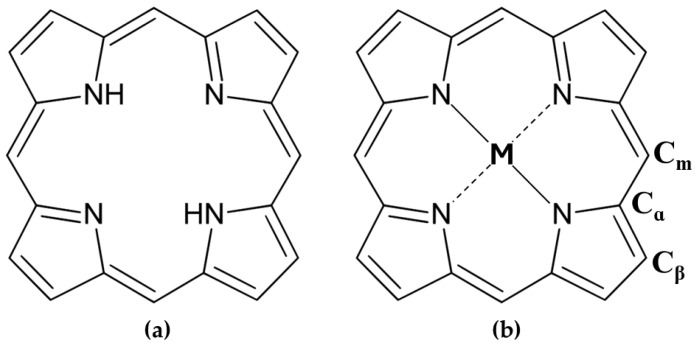
Chemical structures of (**a**) free-base porphyrin and (**b**) metalloporphyrin. A position (Cα); β position (Cβ) and meso position (Cm) represent the three peripheral substituent positions.

The porphyrinic macrocycle (Figure 1a) comprises four pyrrole rings interconnected by methine bridges, forming a planar aromatic system with 22-π electrons, 18 of which are delocalized. This extensive conjugation imparts porphyrins with intense colors and strong absorption in the visible spectrum, contributing to their diverse electronic [3], optical [4,5], mechanical [6], and chemical [7] properties. At the core, the nitrogen atoms create a pocket ideal for coordinating metal ions (e.g., Fe, Co, Mg) in a tetradentate manner [8], which could enable charge transfer and reversible electronic changes, such as oxidation or spin state transitions [1]. Porphyrins can be modified at their peripheral α, β, and meso positions (Figure 1b), allowing for tailored molecular and crystalline properties, including solubility, reactivity, and photophysical behavior. Metalloporphyrins, incorporating transition or non-metal ions, can form various metal–ligand complexes with applications spanning biosensors, phototherapy, and catalysis [9,10].

Due to their unique photophysical and chemical properties, porphyrins are extensively applied in fields such as photoimmunotherapy, photo diagnosis [11], biosensing [12], cancer therapy [13], photocatalysis [14], solar energy conversion [15,16], chemical sensing [17], optoelectronics [18], molecular recognition [19,20,21], and the development of new chiral catalysts for asymmetric synthesis [22,23].

Another particularly intriguing aspect of porphyrins lies in their chiroptical properties which represent the optical properties related to the interaction of chiral structures with circularly polarized light. Chiroptical properties of molecules, aggregates, or biomolecules can be studied by circular dichroism (CD), which is an absorption spectroscopy technique that measures the difference in absorbance of left- and right-circularly polarized light by a chiral molecule. This difference provides information about the molecular structure and conformation of chiral systems, making CD particularly useful for studying the structure of molecules and the superstructures of supramolecular assemblies [24,25,26]. Although porphyrins themselves are typically achiral, unless functionalized with chiral substituents, they can exhibit induced circular dichroism (ICD) upon interaction with chiral template, in particular ICD arises from chiral distortion of the porphyrin symmetry or intermolecular exciton coupling between at least two chirally oriented chromophores [27].

Indeed, porphyrins have also largely been used to investigate the secondary structures of various biomolecules in aqueous solutions, including phospholipids [28], oligopeptides, proteins [29,30,31,32,33,34,35], and polynucleotides [36,37,38,39,40,41]. This review focuses specifically on porphyrinoids as probes for DNA and RNA conformations, with a particular emphasis on their ability to display ICD signals in the Soret region as a result of the interactions with chiral molecules.

Generally, DNA and RNA probes often consist of aromatic chromophores capable of intercalating with nucleic bases via π–π interactions. Among these, porphyrins stand out due to several unique properties:

1. Their highly conjugated electronic system, which produces intense absorption bands in the 380–450 nm region (Soret region), allows their use in the micromolar range, far from the UV region, where most of biomolecules absorb, avoiding spectroscopic interferences.
2. Their structural versatility, allowing functionalization at the meso positions with specific groups or charged moieties to tailor properties such as water solubility.
3. Their ability to coordinate metal ions in the central core, such as zinc or magnesium, which provide additional stereochemical differentiation through Lewis acid sites for binding functional groups like OH, NH_2_, and COO^−^ [42].
4. When non-chiral substituents are present, these molecules are achiral and do not exhibit intrinsic chiroptical signals. However, interactions with chiral molecules could induce CD signals in the Soret absorption band region, which are highly indicative of the binding mode and are far from the UV region where most biomolecules, such as DNA and RNA, absorb [43].
5. Their capacity to act as photosensitizers in the presence of oxygen, enabling their use in photodynamic therapy (PDT) [44,45].

Over the past two decades, extensive research has utilized free-base and metalated porphyrins for both covalent and non-covalent integration into DNA scaffolds. These studies aim to explore porphyrins’ effects on DNA to exploit them as spectroscopic sensors for conformational analysis, as well as to control their stability and electronic properties using techniques such as circular dichroism (CD), CD melting, UV-Vis, fluorescence spectroscopy, and resonance light scattering (RLS) [46]. Since the interactions between porphyrins and chiral polynucleotides could induce a dichroic band (ICD) in the Soret absorption region, CD measurements are particularly precise for diagnosing interaction types. The shape and intensity of ICD signals provide valuable insights into binding mechanisms.

This review highlights the versatility of porphyrins in scientific research and their capacity to address complex challenges in chemical and conformational biological systems. This work further explores their unique chiroptical interactions with nucleic acids, offering valuable insights into molecular dynamics and structural stability.

## 2. Porphyrins as Chiroptical Probes in the Structural Investigation of Nucleic Acids

### 2.1. DNA Polymorphism and Types of Porphyrin Interactions

**Figure 2:**
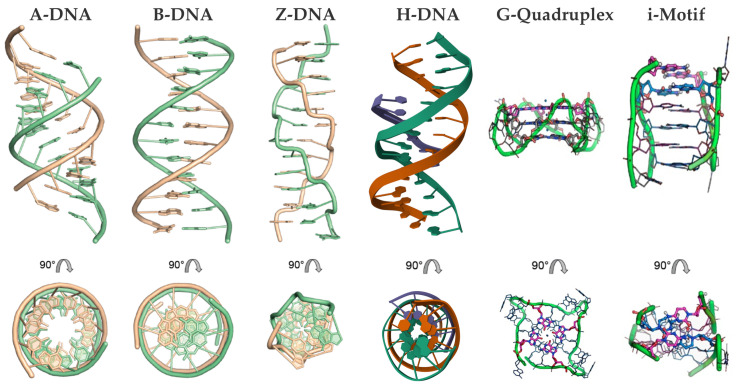
Illustration of various DNA conformations. From left to right: duplex DNA conformations, including right-handed A-form, B-form, and left-handed Z-form, followed by secondary structures such as triplex DNA (H-DNA), and tetraplex structures, including G-quadruplex and i-motif. At the bottom, orthogonal views of each respective structure are shown.

DNA predominantly forms double-helical structures, with its oligonucleotide strands arranged in an antiparallel orientation. The most common conformation is the canonical right-handed B-form; however, depending on the sequence of the bases and environmental conditions, DNA can also adopt other conformations, such as the compact A-form and the left-handed Z-form [47,48,49,50]. Beyond such duplex forms, DNA can form alternative secondary structures, including triplexes, G-quadruplexes, and i-motifs, due to alternative base pairing (e.g., Hoogsteen or reverse Hoogsteen interactions) and π–π stacking (Figure 2) [51,52,53,54,55,56].

These structures are implicated in various biological processes, including the regulation of gene expression [57]. For instance, specific sequences and structural motifs of nucleic acids can be exploited for understanding the mechanisms of genetic diseases and developing targeted treatments [50,51,55,58,59,60]. Additionally, the interactions with natural and synthetic molecules, among them aromatic chromophores, are central to regulating genetic processes [61,62,63]. These small molecules play critical roles in therapeutic applications, such as anticancer and antibiotic treatments, gene expression regulation, photodynamic therapy, and structural studies of DNA, by binding through intercalation, groove binding, or covalent interactions to alter or probe DNA structure and function [62].

Porphyrins stand out among these small molecules as a remarkable class of compounds highly effective in interacting with DNA. Their ability to bind nucleic acids through diverse mechanisms further highlights their potential as probes and stabilizers of DNA structures.

Fiel et al. (1979) [64] and Fiel and Munson (1980) [65] were the first to provide an extensive analysis of nucleic acid interactions with meso-5,10,15,20-tetrakis-(*N*-methyl-4-pyridyl)porphine (H_2_TMPyP4) and its metal derivatives (FeTMPyP4, CoTMPyP4, NiTMPyP4, ZnTMPyP4, SnTMPyP4, MnTMPyP4).

A few years later, in 1983, Pasternack et al. [66] explained the structural properties of metalloporphyrins, emphasizing how factors such as the type of metal ion, and the size and positioning of substituents, significantly influence their binding with polynucleotides. Several parameters, including the porphyrin’s charge, peripheral substituents, solvent environment, ionic strength, and the porphyrin-to-DNA ratio, play a crucial role in modulating these interactions; furthermore, by altering the metal core and substituents, the binding modes can be easily changed [67].

**Figure 3:**
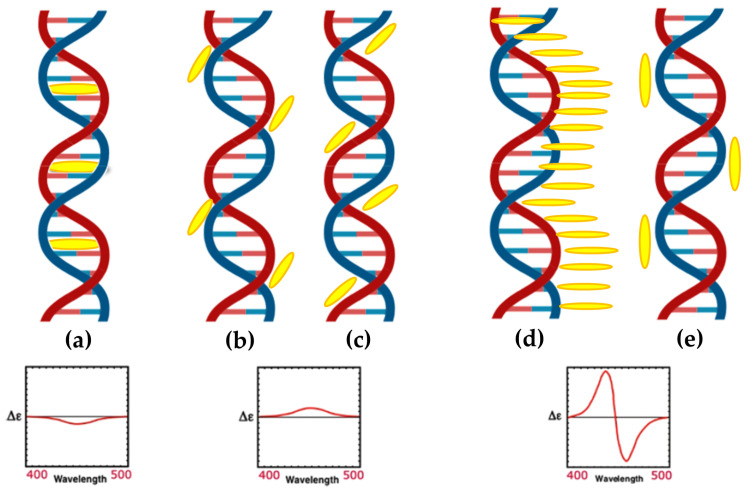
Schemes of various binding modes of porphyrins to DNA. (**a**) Intercalation, (**b**) binding to the minor groove of DNA, (**c**) binding to the major groove of DNA, (**d**) external binding with self-stacking along the DNA surface, and (**e**) long-range porphyrin–porphyrin interactions. At the bottom, induced CD signals in the Soret region corresponding to each binding mode are shown.

Porphyrins interact with double-stranded DNA through multiple mechanisms, including intercalation, groove binding (in both minor and major grooves), and external binding (outside stacking) (Figure 3) [68].

Fiel and colleagues [69] referred to the first three mechanisms collectively as a ’three-mode binding model.’ Intercalation occurs when the planar aromatic porphyrin ring is introduced between two base pairs, leading to DNA unwinding and elongation, which is usually characterized by a negative induced CD signal in the Soret region (Figure 3a). Groove binding, on the other hand, involves electrostatic interactions between the cationic porphyrin and the negatively charged phosphate backbone, with A–T base pairs favoring minor groove binding and G–C base pairs often forming intercalated complexes. Parameters such as ionic strength and the porphyrin-to-base-pair ratio strongly influence the competition between intercalation and groove binding [70]. Regarding groove binding a small positive or even no induced CD is observed (Figure 3b,c) [37]. The third binding mechanism known as outside stacking can include an ordered aggregation of the porphyrins, which interact with each other through π–π interactions and electrostatic interactions along the external backbone of the DNA helix. This arrangement usually produces intense induced bisignate CD signals (Figure 3d) [37]. Outside stacking can also include the interactions of a few porphyrins organized outside the DNA helix that do not interact directly with each other. Even in this case, an induced bisignate CD signal in the Soret region can be observed, although with lower intensity, as the ICD originates from long-range exciton coupling between different porphyrins bound to the DNA helix (Figure 3e) [71].

**Figure 4:**
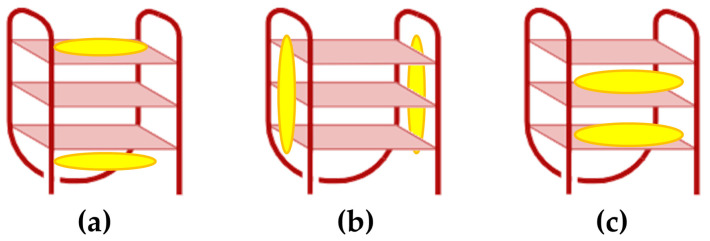
Illustrations of various binding modes of porphyrins to DNA G4. (**a**) Capping at the ends of the quadruplex, (**b**) external binding along the quadruplex strands, and (**c**) intercalation between guanine tetrads within the G4 structure.

For G-quadruplexes (G4), porphyrin interactions are shaped by several structural and sequence-specific factors, including the type of G4, strand orientation, loop configuration, groove dimensions, and adjacent non-G-tetrad elements [72]. These interactions occur through three primary modes: (i) capping at the ends of the quadruplex [73], (ii) binding externally along the quadruplex strands [74], and (iii) intercalating between guanine tetrads within the G4 structure (Figure 4) [75].

In the following subsections, the interactions between porphyrins and various DNA structures will be discussed, with a particular emphasis on the induced chiroptical signals observed in the visible region. The reported works primarily focus on studies published over the past decade, as the interactions between porphyrins and DNA structures were comprehensively described by our group in earlier book chapters published in 2013 [68] and 2014 [72], respectively.

#### 2.1.1. Chiroptical and Structural Insights into Porphyrin Interactions with B-DNA

**Figure 5:**
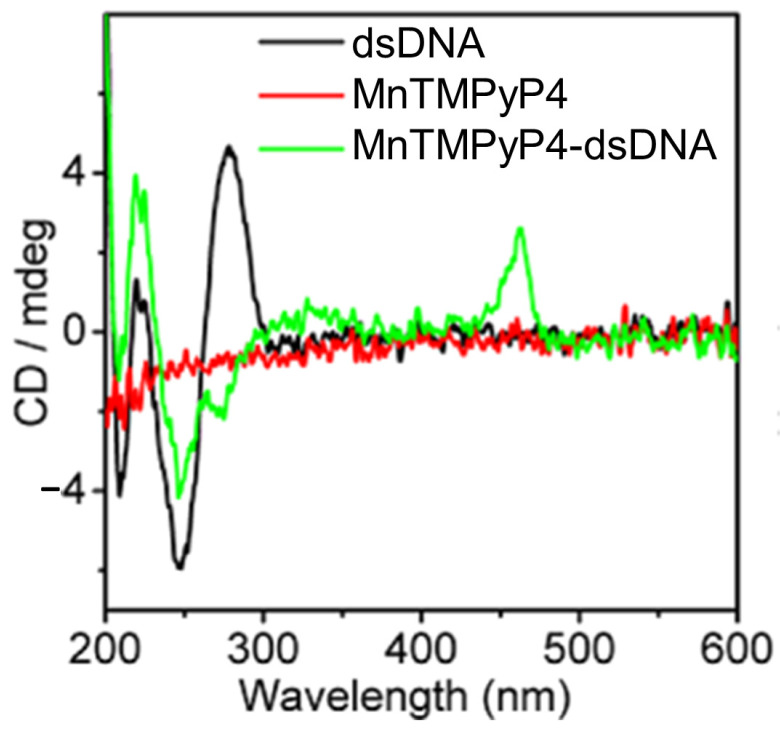
CD spectra of 5.0 μM double-stranded DNA (dsDNA) (black line), 100 μM MnTMPyP4 (red line), and a mixture containing 5.0 μM dsDNA with 90 μM MnTMPyP4 (green line). Adapted with permission from ref. [76]. Copyright 2013 American Chemical Society.

In 2013, Jie Xu and colleagues [76] introduced a manganese(III) porphyrin-dsDNA complex (MnTMPyP4-dsDNA) as a highly efficient peroxidase-mimicking enzyme with significant bioanalytical applications. MnTMPyP4 was shown to bind double-stranded DNA (dsDNA) through groove binding, confirmed by the induced CD signal at 462 nm (Figure 5) and a hyperchromic shift in the Soret band. The interaction did not disrupt the dsDNA structure, and the binding constant (K ≈ 1.58 × 10^5^ M^−^^1^) indicated a strong association of MnTMPyP4 to dsDNA. This complex was also utilized for chemiluminescent (CL) bioanalysis, demonstrating amplified sensitivity through a hybridization chain reaction (HCR). The strategy combined the catalytic properties of MnTMPyP4 with DNA scaffolding, achieving a detection limit of 6.8 pg/mL for carcinoembryonic antigen (CEA), outperforming traditional enzyme labels. The MnTMPyP4-dsDNA complex represents a promising advance in bioanalytical applications, offering a robust and versatile alternative to natural enzyme systems.

**Figure 6:**
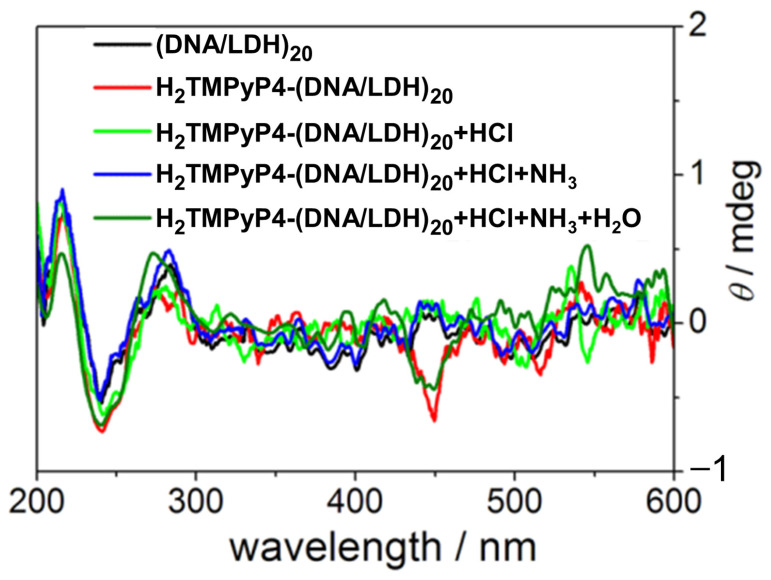
CD spectra of (DNA/LDH)_20_ (black line), H_2_TMPyP4-(DNA/LDH)_20_ (red line), H_2_TMPyP4-(DNA/LDH)_20_ after exposure to HCl (green line), and NH_3_/H_2_O vapor (dark green line). Adapted with permission from ref. [77]. Copyright 2014 American Chemical Society.

In 2014, Shi et al. [77] investigated the chiroptical properties and interaction mechanisms of H_2_TMPyP4 with B-DNA immobilized within a layered double hydroxide (LDH) matrix denoted as H_2_TMPyP4-(DNA/LDH)_20_. H_2_TMPyP4 produced a negatively induced CD band at 449 nm (Figure 6) upon intercalation into the DNA base pair cavities, highlighting the specific orientation and supramolecular chiral environment created within the LDH matrix.

The intercalation occurred in two distinct steps: initial electrostatic adsorption to the DNA/LDH matrix surface, followed by thermal activation at 70 °C, which promoted deeper intercalation into the DNA helix evidenced by the appearance of the negative ICD. Furthermore, the study demonstrated the reversible nature of H_2_TMPyP4 intercalation, with a deintercalation process with HCl vapor and subsequent NH_3_/H_2_O vapor restoring it, evidenced by the reappearance of the ICD signal (Figure 6, dark green line). This reversibility suggests the potential application of TMPyP in chiroptical switches and molecular sensing devices.

**Figure 7:**
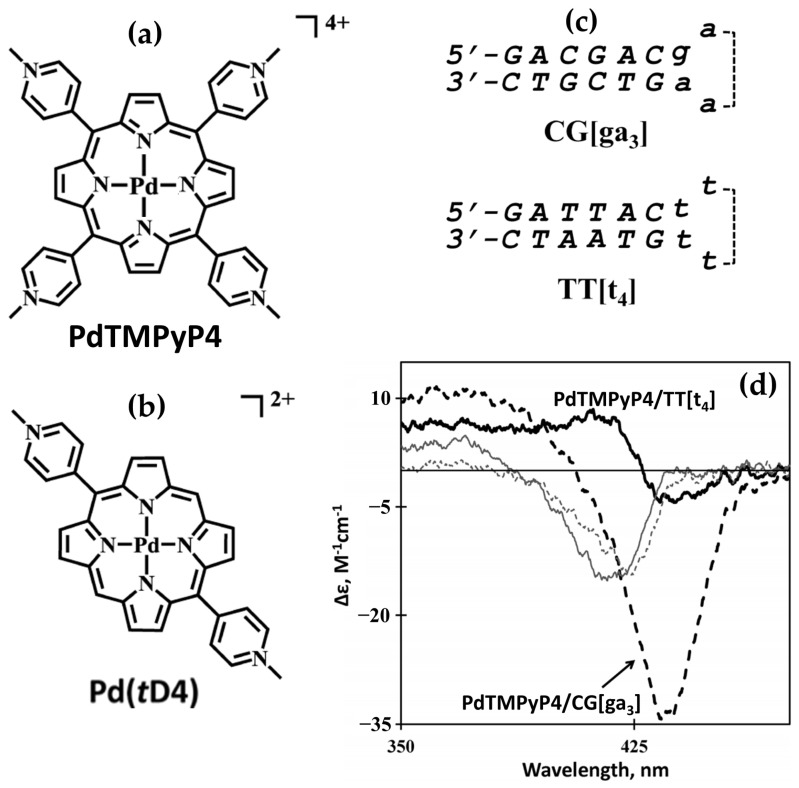
Structures of (**a**) [5,10,15,20-tetra(4-*N*-methylpyridyl)]palladium(II) (PdTMPyP4) and of (**b**) [5,15-di(4-N-methylpyridyl)porphyrin]palladium(II) (Pd(tD4)). (**c**) DNA sequences used in the work: CG[ga3], 5′-GACGACgaaaGTCGTC-3′; TT[t4], 5′-GATTACttttGTAATC-3′. (**d**) Induced CD spectra of PdTMPyP4 interacting with DNA hairpins TT[t4] (thick solid line) and CG[ga3] (thick dashed line); and Pd(tD4) bound to TT[t4] (thin solid line) and CG[ga3] (thin dashed line). Adapted with permission from ref. [78]. Copyright 2014 American Chemical Society.

**Figure 8:**
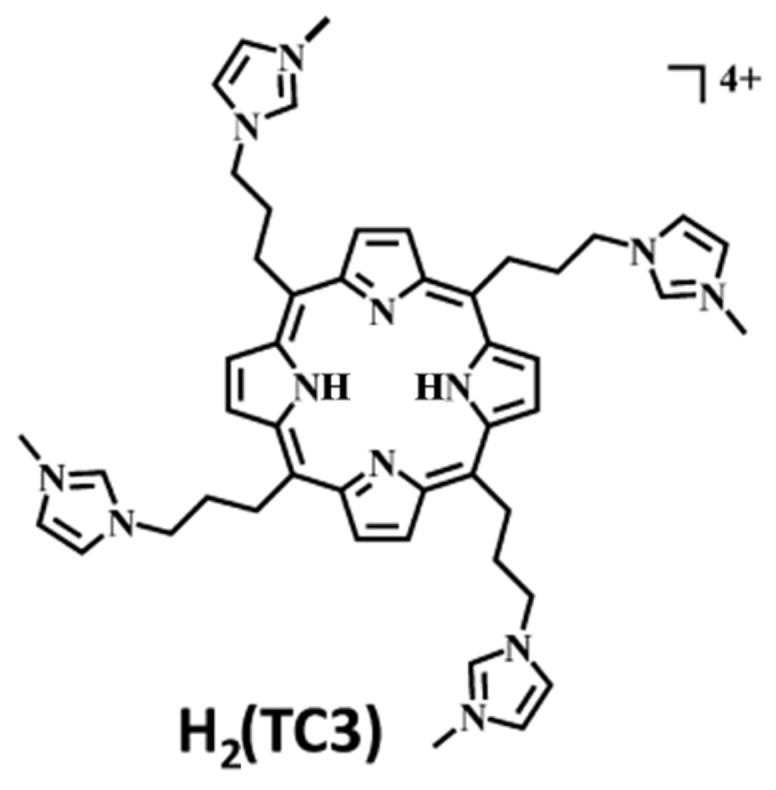
Structure of 5,10,15,20-tetra [3-(3′-methylimidazolium-1′-yl)]-porphyrin (H_2_(TC3)). Adapted with permission from ref. [79]. Copyright 2014 American Chemical Society.

In 2014, the group led by McMillin explored the interactions of palladium(II)-containing cationic porphyrins, PdTMPyP4 (Figure 7a) and [5,15-di(4-*N*-methylpyridyl)porphyrin]palladium(II), or Pd(tD4) (Figure 7b) [78] and a tetraalkyl-substituted cationic porphyrin, H_2_TC3 (Figure 8), along with its metal derivatives Cu(TC3) and Zn(TC3) [79], with double-stranded DNA, revealing how structural variations influence binding modes and chiroptical responses. PdTMPyP4, with its sterically hindered structure, exhibited dual binding modes: intercalation with G≡C-rich sequences, characterized by hypochromism and bathochromic shifts in the Soret band and negative induced CD signals. Whereas with A=T-rich sequences, it produces bisignate ICD signals indicative of groove binding. In contrast, Pd(tD4), with reduced steric hindrance, exclusively intercalated into DNA, evidenced by consistently negative ICD signals and enhanced spectral shifts, highlighting the critical role of steric factors in modulating binding preferences.

Similarly, H_2_TC3 (Figure 8) displayed sequence-dependent binding modes. With G≡C-rich B-DNA, it predominantly intercalated, producing negative ICD signals and bathochromic shifts in the Soret band. However, with A=T-rich sequences, H_2_TC3 favored external binding, generating positive ICD signals. The Cu(TC3) derivative exclusively intercalated into DNA, irrespective of sequence, demonstrating strong π–π stacking interactions, while Zn(TC3) showed a preference for external binding due to its axial ligand coordination, leading to biphasic, predominantly positive ICD signals and weaker spectral shifts compared to Cu(TC3).

Photophysical studies further distinguished these porphyrins. Intercalation by Pd(tD4) and Cu(TC3) extended triplet-state lifetimes, enhancing singlet oxygen generation essential for photodynamic therapy, while external binding, as observed for PdTMPyP4 and Zn(TC3), provided structural stabilization without achieving comparable sensitization. These findings underscore the interplay between steric factors, metal centers, and substituent flexibility in determining DNA binding modes and functional results, offering valuable understandings for designing porphyrin-based probes and therapeutic agents targeting DNA structures.

**Figure 9:**
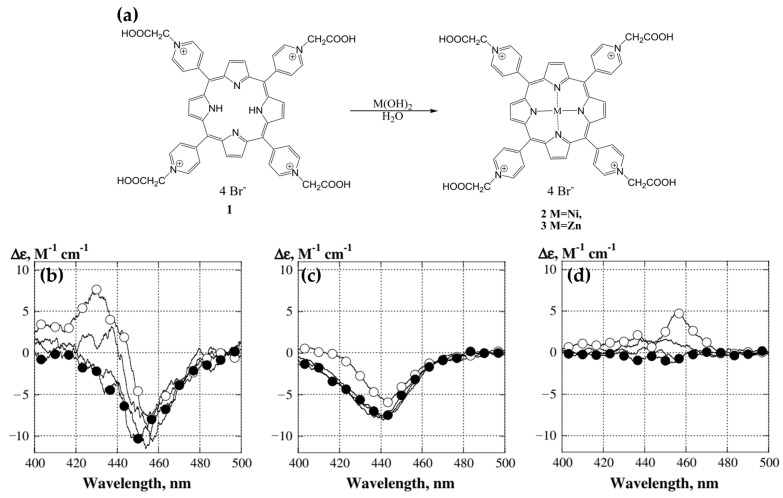
(**a**) Preparation of metal derivatives of 5,10,15,20-tetrakis(*N*-carboxymethyl-4-pyridinium)porphyrin. Circular dichroism spectra of DNA:porphyrin complexes in the Soret band region for (**b**) P1, (**c**) NiP1, and (**d**) ZnP1. The CD spectra of DNA:porphyrin complexes are represented by open circles, with porphyrin concentrations ranging from 1 to 3 μM and ct-DNA at 30 μM (bp). Addition of distamycin A led to displacement of ZnP1 and P1 from minor groove binding. Distamycin A concentrations were as follows: unmarked curves correspond to 1 and 2 μM, while filled circles represent 3 μM. Adapted with permission from ref. [80]. Copyright 2014 European Biophysics Journal.

In 2014, Kovaleva and coworkers [80] investigated the interactions of 5,10,15,20-tetrakis(N-carboxymethyl-4-pyridinium)porphyrin (P1) and its metal derivatives, ZnP1 and NiP1 (Figure 9a), with calf thymus DNA (ct-DNA). Their study provided key insights into the binding mechanisms and associated chiroptical properties of these porphyrins. CD spectra revealed that ZnP1 predominantly binds to the DNA minor groove, producing a positive induced CD signal in the Soret region (Figure 9d), whereas NiP1 intercalates into the DNA double helix, giving rise to a negative induced CD signal (Figure 9c). The metal-free P1, on the other hand, exhibited both groove-binding and intercalative modes, indicated by a superimposed CD spectrum featuring both positive and negative bands (Figure 9b).

These interactions were further confirmed through competitive displacement assays with distamycin A, a known minor groove binder. Distamycin A effectively displaced ZnP1 from the DNA groove, reducing the positive CD signal (Figure 9d, filled circles), while leaving the intercalated NiP1 largely unaffected. Docking simulations supported these findings, illustrating ZnP1′s groove-binding preference, facilitated by its square pyramidal geometry and additional axial coordination. In contrast, NiP1′s square planar geometry favored intercalation between DNA base pairs.

The differential binding modes also impacted the DNA photodamaging potencies of these porphyrins. ZnP1 exhibited the highest efficiency in generating singlet oxygen and inducing plasmid DNA photocleavage, significantly outperforming P1 and NiP1. This superior activity was attributed to ZnP1’s ability to localize in the groove, facilitating reactive oxygen species (ROS) generation near DNA, in contrast to the solvent-shielded intercalative complex of NiP1.

**Figure 10:**
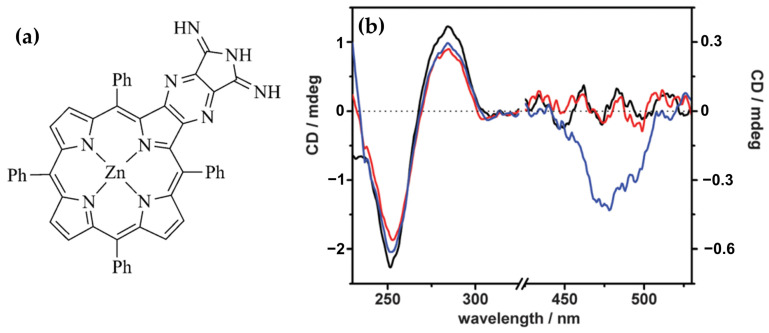
(**a**) Structure of the zinc diiminoisoindoline–porphyrin (compound **3**). (**b**) CD spectra of a 10 µM poly(dG-dC) solution in 1 mM cacodylate buffer (black line) with addition of 4 µM compound **3** (red line), following thermal treatment by heating to 90 °C and rapid cooling to 25 °C (blue line). Adapted with permission from ref. [81]. Copyright 2016 Royal Society of Chemistry.

In 2016, Mandoj et al. [81] explored the interaction of a novel β-fused isoindoline–porphyrin conjugate (compound **3**) (Figure 10a) with polynucleotides, revealing unique binding mechanisms and induced chiroptical properties. Initial experiments indicated no significant interaction between compound **3** and poly(dG-dC) under standard conditions, as confirmed by UV/Vis and CD spectra. This lack of interaction was attributed to the absence of positive charges on the porphyrin, limiting electrostatic attraction to the negatively charged DNA backbone, and the steric hindrance caused by the axial ligand, which prevented intercalation. However, upon thermal treatment—heating the DNA–porphyrin mixture to 90 °C and rapidly cooling it—compound **3** successfully intercalated into the DNA double helix. The resulting CD spectra exhibited a negative ICD signal in the Soret region (Figure 10b), accompanied by an 8 nm red-shift, characteristic of intercalative binding. Notably, the interaction did not perturb the overall stability of the poly(dG-dC) double helix, even after one hour, highlighting the non-disruptive nature of the binding. Compared to conventional porphyrins that rely on positive charges for DNA interaction, the β-fused isoindoline modification facilitated intercalation through thermal induction, underscoring the influence of structural modifications on DNA affinity. The unique ICD signal observed in this study suggests that compound **3** can serve as an effective chiroptical probe for intercalative binding, with potential applications in drug delivery systems using DNA as a scaffold. Additionally, the incorporation of the isoindoline unit enhances hydrogen-bonding capabilities, making this porphyrin derivative a promising candidate for supramolecular and biomedical applications.

**Figure 11:**
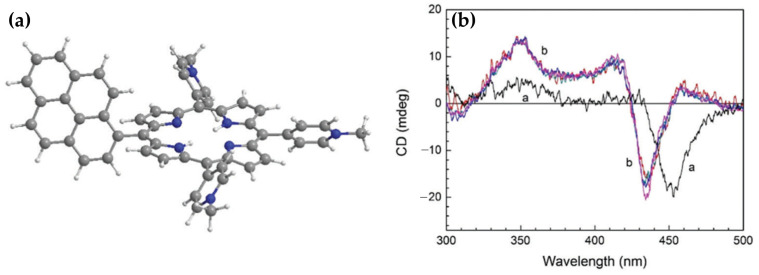
(**a**) Chemical structure of (1-pyrenyl)-tris(N-methyl-p-pyridino)-porphyrin (PyTMpyP). (**b**) CD spectra of TMpyP (curve a) and PyTMpyP (curve b) in complex with DNA. Spectra were recorded at [PyTMpyP]/[DNA] ratios of 0.04, 0.06, 0.08, and 0.10. For TMpyP, the absorption spectrum at [TMpyP]/[DNA] = 0.05 was scaled by a factor of 2. DNA concentration was maintained at 100 μM. Adapted with permission from ref. [82]. Copyright 2017 Royal Society of Chemistry.

In 2017, Lee et al. [82] focused on the interaction between a pyrene–porphyrin dyad, (1-pyrenyl)-tris(N-methyl-p-pyridino)porphyrin (PyTMpyP) (Figure 11a), and DNA, with an emphasis on the photophysical and chiroptical properties resulting from this interaction. CD spectroscopy revealed unique features in the Soret and pyrene absorption regions. The PyTMpyP-DNA complex exhibited a bisignate CD signal in the Soret region with two positive peaks at 414 and 459 nm and a negative peak at 435 nm (Figure 11b). These features differ markedly from those of the parent TMpyP-DNA complex, which displayed a simple negative CD band, indicative of intercalation. The additional positive bands in the PyTMpyP-DNA spectrum suggest complex interactions influenced by the pyrene moiety.

In the pyrene absorption region (300–360 nm), the PyTMpyP-DNA complex displayed a strong positive CD band at ~350 nm (Figure 11b), suggesting minor groove binding of the pyrene moiety. The perpendicular orientation of the pyrene and porphyrin moieties in aqueous solution and the absence of stacking interactions were supported by the lack of a classical intercalation signal in linear dichroism (LD) experiments. Instead, the data indicated that PyTMpyP binds externally to the DNA, causing significant alterations in DNA conformation.

The study highlighted a photoinduced electron transfer (PET) process between the pyrene and porphyrin moieties. In aqueous solution, the PET process quenched the fluorescence of PyTMpyP. However, upon DNA binding, PET was suppressed, enhancing porphyrin fluorescence and enabling singlet oxygen generation. These findings underline the critical role of the binding mode in modulating the photophysical properties of PyTMpyP-DNA complexes, providing insights into its potential as a photodynamic therapy agent and a chiroptical probe for DNA interactions.

**Figure 12:**
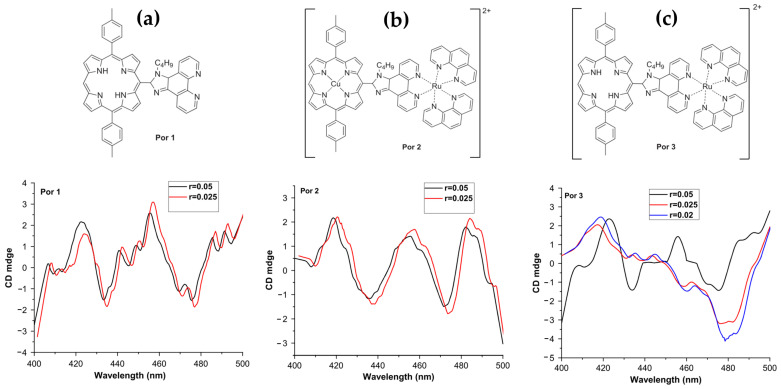
Top panel: Chemical structures of porphyrin derivatives (**a**) **Por 1**, (**b**) **Por 2**, and (**c**) **Por 3**. Bottom panel: Corresponding induced CD spectra of **Por 1–3** bound to ct-DNA ([porphyrin] = 10 μM) in buffer solution (pH 7.4; 0.05 M Tris–HCl, 0.1 M NaCl), with [porphyrin]/[DNA base pair] ratios of r = 0.05, 0.025, and 0.02. Adapted with permission from Ref. [83]. Copyright 2018 John Wiley and Sons.

Jiang et al. in 2018 [83], synthesized three porphyrin derivatives—**Por 1** (a conjugated porphyrin–imidazo [4,5-f]phenanthroline ligand), **Por 2** (its ruthenium-containing complex), and **Por 3** (a free-base porphyrin derived from **Por 2**) (Figure 12a–c, top panel)—to investigate their binding modes with DNA. Using CD, the authors revealed distinct interaction mechanisms for these compounds with ct-DNA. **Por 1** exhibited a strong positive CD signal at 410 nm and a weaker peak at 433 nm, indicative of an external binding mode (Figure 12a, bottom panel). Conversely, **Por 2** and **Por 3** showed negative CD signals at 433/481 nm (Figure 12b, bottom panel) and 479 nm (Figure 12c, bottom panel), respectively, which were consistent with intercalative binding.

The CD data highlighted a progression in binding interactions, with **Por 2** and **Por 3** demonstrating stronger affinity for intercalative modes due to the introduction of a cationic ruthenium complex. This positive charge facilitated stable electrostatic interactions with the negatively charged DNA phosphate backbone. Additionally, the binding constants calculated for **Por 1**, **Por 2**, and **Por 3** were 7.79 × 10^3^, 1.29 × 10^4^, and 1.32 × 10^4^ M^−^^1^, respectively, further supporting the enhanced DNA affinity of the ruthenium-containing derivatives.

This work underscores the potential of porphyrin derivatives as tools for DNA interaction studies, showcasing how structural modifications—like introducing metal centers—can tune binding affinities and types of interaction.

**Figure 13:**
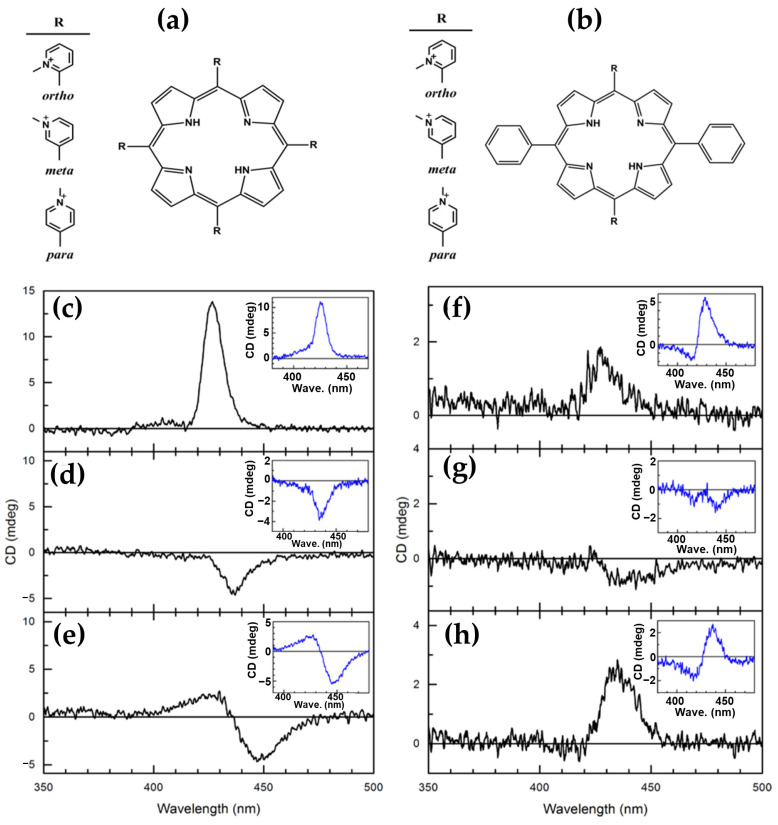
Chemical structures of (**a**) H_2_TMPyP derivatives—ortho (H_2_TMPyP2), meta (H_2_TMPyP3), and para (H_2_TMPyP4)—and (**b**) *trans*-bis(N-methylpyridiniumyl) diphenyl porphyrins—ortho (*trans*-BMPyP2), meta (*trans*-BMPyP3), and para (*trans*-BMPyP4). CD spectra of (**c**) H_2_TMPyP2, (**d**) H_2_TMPyP3, and (**e**) H_2_TMPyP4 bound to DNA under molecular crowding conditions, with corresponding spectra in aqueous solution shown in insets. CD spectra of (**f**) *trans*-BMPyP2, (**g**) *trans*-BMPyP3, and (**h**) *trans*-BMPyP4 bound to DNA in PEG solution, with their spectra in aqueous solution displayed in insets. [DNA] = 100 μM; [BMPyP] = 10 μM. Adapted from ref. [84]. Published by American Chemical Society, 2020; further permissions related to the material excerpted should be directed to the ACS.

In the same year, Cho and coworkers [84] investigated the interactions of various cationic porphyrins with DNA under molecular crowding conditions induced by poly(ethylene glycol) (PEG). Three H_2_TMPyP derivatives, ortho (H_2_TMPyP2), meta (H_2_TMPyP3), and para (H_2_TMPyP4) (Figure 13a) and their *trans*-bis(*N*-methylpyridiniumyl) diphenyl analogs (*trans*-BMPyPs) (Figure 13b) were examined using CD and linear dichroism (LD) spectroscopy.

In aqueous solution, distinct binding modes were observed among the porphyrins. H_2_TMPyP3 and H_2_TMPyP4 predominantly intercalated into DNA, as evidenced by strong negative CD signals in the Soret region (Figure 13d,e), consistent with stacking between DNA base pairs. Conversely, H_2_TMPyP2 displayed a positive CD signal (Figure 13c), indicative of groove or external binding to DNA. LD spectra corroborated these findings, with H_2_TMPyP3 and H_2_TMPyP4 exhibiting pronounced negative signals characteristic of intercalation, while H_2_TMPyP2 showed weaker signals, supporting major groove binding.

Under molecular crowding conditions created by PEG, the binding modes of H_2_TMPyPs remained largely unaffected. CD spectral features were preserved (Figure 13c–e, inset), indicating that PEG does not disrupt intercalated or groove-bound porphyrins and does not penetrate DNA. This stability under crowded conditions underscores the robustness of H_2_TMPyP-DNA interactions.

In contrast, *trans*-BMPyPs exhibited more complex behavior. Their CD spectra in aqueous solution (Figure 13f–h, inset) deviated from those of H_2_TMPyPs, suggesting a different binding mechanism, likely external binding to the DNA surface. Under PEG-induced crowding, the CD of *trans*-BMPyPs undertook significant changes (Figure 13f–h), reflecting altered interactions with DNA. These findings imply that molecular crowding selectively influences *trans*-BMPyPs, likely due to their greater accessibility to the PEG matrix.

This study highlights the differential effects of molecular crowding on DNA–porphyrin interactions. While H_2_TMPyPs demonstrate stable intercalative or groove-binding modes, *trans*-BMPyPs exhibit sensitivity to crowded environments, suggesting potential applications in environments where molecular crowding plays a critical role, such as cellular contexts.

**Figure 14:**
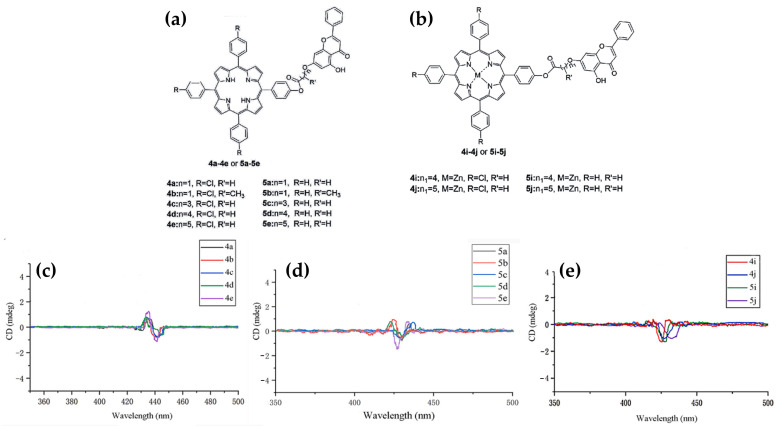
Chemical structures of the porphyrin–chrysin derivatives (**a**) without a metal in central core and (**b**) with Zn in central core. ICD spectra of porphyrin–chrysin derivatives (**c**) **4a**–**4e**, (**d**) **5a**–**5e**, and (**e**) **4i**–**5j** interacting with ct-DNA at a porphyrin-to-ct-DNA molar ratio of 0.1. Samples were prepared in Tris-HCl buffer with ct-DNA concentration of 100 µM. Adapted with permission from ref. [85]. Copyright 2023 Elsevier.

In 2023, Zhang et al. [85] explored the synthesis, antitumor activity, and DNA binding interactions of novel porphyrin–chrysin derivatives (Figure 14a,b), emphasizing their potential as photosensitive drugs for photodynamic therapy (PDT) applications. The study revealed that the interaction between porphyrins and ct-DNA was highly dependent on the structural features of the porphyrins. CD studies indicated that free-base porphyrin derivatives (**4a**–**4e**, **5a**–**5e**) (Figure 14a) interact with ct-DNA via surface self-stacking, as evidenced by alternating positive and negative induced CD signals in the Soret region (400–450 nm) (Figure 14c,d). These interactions were attributed to groove binding or stacking with multiple possible ligand orientations. Conversely, zinc metalloporphyrin derivatives (**4i**, **4j**, **5i**, **5j**) (Figure 14b) exhibited a prominent negative ICD signal in the Soret region (Figure 14e), indicative of intercalation, which was further supported by UV-Vis absorption and fluorescence quenching experiments.

The study also highlighted a strong correlation between DNA binding tightness and antitumor activity. Free-base derivatives displayed stronger inhibitory effects on HeLa and A549 cancer cells under light conditions compared to their zinc counterparts, demonstrating their photodynamic efficacy. The findings underscore the significance of porphyrin structural modifications, such as introducing electron-withdrawing groups or increasing the charge density, to enhance their DNA binding and therapeutic potential.

#### 2.1.2. Porphyrin Derivatives as Probes for the Z-DNA

Z-DNA, first identified in 1972 [68], is a left-handed, high-energy conformation of double-stranded DNA that has intrigued researchers due to its distinct structure and potential biological significance [50,86,87,88]. Characterized by a zigzag pattern in the sugar–phosphate backbone, Z-DNA features 12 base pairs per helical turn. Its guanine residues adopt a *syn* conformation, while cytosine residues remain in an *anti* conformation. This configuration eliminates the traditional major groove, leaving only a reversed minor groove accessible for interactions (Figure 2) [89].

The transition from the canonical right-handed B-DNA to Z-DNA is highly sequence- and condition-dependent, typically occurring in alternating pyrimidine–purine sequences under high concentrations of cations such as Na^+^, Ni^2+^, or Co(NH_3_)_6_^3+^, or in the presence of polycationic amines like spermine^4+^ [90]. These transitions can be monitored using CD spectroscopy, which shows distinct spectral features for each form: B-DNA exhibits a positive band at 280 nm and a negative band at 250 nm, while Z-DNA displays a negative band at 290 nm and a positive band at 260 nm [91].

Although the biological role of Z-DNA remains partially understood, evidence suggests it may play a role in transcriptional regulation [88,92,93]. Certain proteins with positively charged side chains are capable of selectively recognizing and stabilizing Z-DNA, creating an environment conducive to the B-to-Z transition [50]. While intracellular conditions may not naturally support this transition in unmodified DNA, associations between Z-DNA and neurological disorders or autoimmune diseases, such as those linked to aluminum-induced Z-DNA formation, have been reported [94]. However, its detection and study remain challenging due to low B/Z DNA ratios and spectroscopic interferences in biological systems [95,96,97].

Recent studies have extensively explored the interactions between porphyrins and DNA, focusing on their ability to induce and stabilize conformational changes, particularly the B–Z transition.

**Figure 15:**
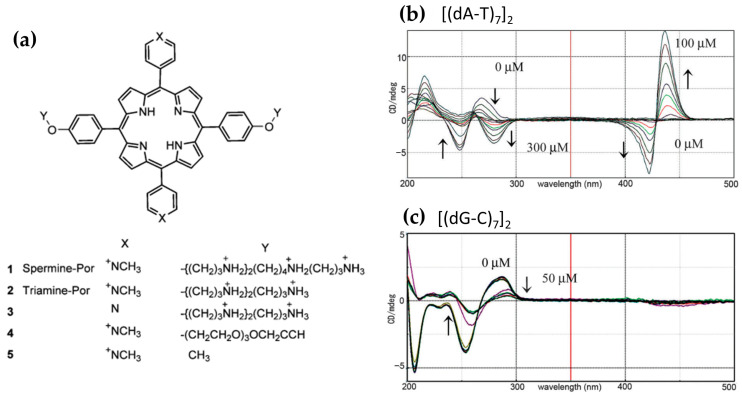
(**a**) Structure of porphyrin derivatives (1–5) used. CD spectral changes observed during titration of compound **1** into a solution of 15 mM [(dA-T)_7_]_2_ (**b**) and [(dG-dC)7]2 (**c**) in a buffer containing 1 mM sodium cacodylate and 100 mM NaCl, at pH = 7.0 and 20 °C. Arrows indicate increases or decreases in CD bands upon addition of compound **1**. Adapted with permission from ref. [98]. Copyright 2013 Royal Society of Chemistry.

In 2013, Sasaki et al. [98] investigated a spermine–porphyrin derivative’s ability (Figure 15a) to promote the transition from B-DNA to Z-DNA in alternating adenine–thymine duplex sequences [(dA-T)_n_]_2_. The results demonstrated that the porphyrin successfully promoted the Z-DNA conformation in [(dA-T)_7_]_2_, as evidenced by a CD signal with a positive band at 260 nm and a negative band at 280 nm (Figure 15b), which are characteristic of Z-DNA. In contrast, no Z-DNA induction was observed for guanine–cytosine [(dG-C)_7_]_2_ duplexes, where the porphyrin exhibited intercalative binding, retaining the B-form CD signature (Figure 15c).

Further analysis revealed that a related triamine–porphyrin (Figure 15a) exhibited similar B–Z induction selectivity for [(dA-T)_7_]_2_ but with slightly reduced effectiveness compared to the spermine–porphyrin. Porphyrin derivatives lacking either the polyamine or pyridinium cations (Figure 15a) were unable to induce the B–Z transition, confirming the essential role of the conjugated porphyrin structure and the polyamine groups in driving the conformational change. Additionally, no Z-DNA induction was observed for [(dG-C)_n_]_2_ with any porphyrin derivatives. While zinc(II) and copper(II) derivatives of H_2_TMPyP4 are known to bind duplex DNA, the corresponding zinc(II) or copper(II) complexes of 1 and 2 (Figure 15a) did not produce significant changes in the CD spectra, further emphasizing the specificity of the observed B–Z transition for certain porphyrin structures.

CD intensity measurements of [(dA-T)_7_]_2_ with spermine–porphyrin in the Soret band region (Figure 15b) indicated that the transition to Z-DNA was associated with stacked aggregation of the porphyrins at higher ligand-to-DNA ratios. Specifically, no significant changes in the Soret band were observed up to a ratio of 3, after which both positive and negative bands became more pronounced at higher ligand concentrations. This strongly induced CD intensity in the Soret region is consistent with aggregation, which may play a critical role in stabilizing the Z-DNA conformation.

**Figure 16:**
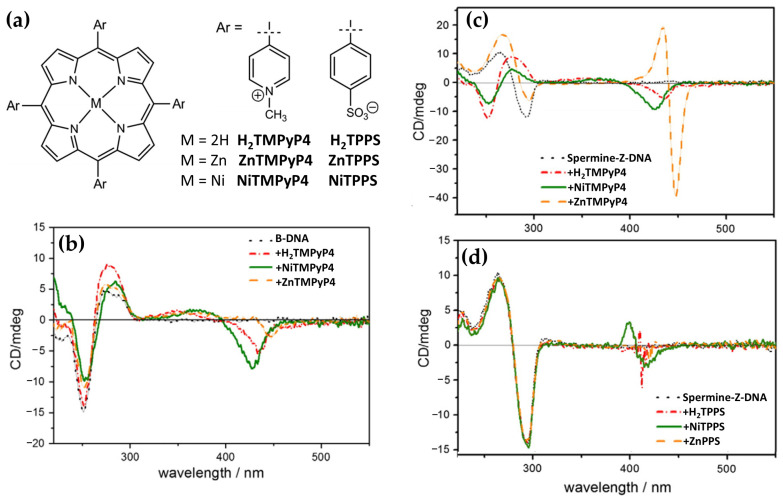
Structures of cationic tetrapyridium (metallo)porphyrins (MTMPyP4) and anionic tetrasulfonated (metallo)porphyrins (MTPPS) (**a**). CD spectra of H_2_TMPyP4 (red dot-dashed line), NiTMPyP4 (green solid line), and ZnTMPyP4 (orange dashed line) recorded in presence of (**b**) B-form of poly(dG-dC)_2_ (50 μM) and in presence of (**c**) Z-form of poly(dG-dC)_2_ (50 μM) induced by spermine (12 μM) in a Na-cacodylate buffer (1 mM, pH 7.0, 10 mM NaCl), with a porphyrin concentration of 6 μM. (**d**) CD spectra of H_2_TPPS (red dot-dashed curve), NiTPPS (green solid curve), and ZnTPPS (orange dashed curve) in presence of Z-form of poly(dG-dC)_2_ (50 μM) induced by spermine (12 μM). Adapted with permission from ref. [43]. Copyright 2013 Elsevier.

In the same year, Kyu Choi and coworkers [43] investigated the interactions with B-DNA and Z-DNA exploiting the chiroptical properties of various porphyrins, including H_2_TMPyP4 and its nickel (NiTMPyP4) and zinc (ZnTMPyP4) derivatives, as well as anionic tetrasulfonatophenyl porphyrins (H_2_TPPS, NiTPPS, ZnTPPS) (Figure 16a). CD spectra revealed distinct binding modes and induced CD signals depending on the porphyrin type and DNA conformation. For B-DNA, cationic porphyrins (H_2_TMPyP4 and NiTMPyP4) exhibited strong negative CD bands in the Soret region (Figure 16b), indicating intercalative binding between the DNA base pairs. ZnTMPyP4, on the other hand, produced a weaker negative CD signal (Figure 16b), suggesting pseudo-intercalation, because of its axially water molecule pentacoordinated that prevents efficient intercalation. In contrast, anionic porphyrins showed negligible CD signals, reflecting weak or absent interactions with the B-form, likely due to electrostatic repulsion. For Z-DNA, ZnTMPyP4 generated strong bisignate CD signals in the Soret region, indicative of stacking interactions along the left-handed helical structure. ZnTMPyP4 exhibited selective groove binding, stabilizing the Z-DNA conformation, while NiTMPyP4 and H_2_TMPyP4 produced a negative induced CD and partially induced a Z-to-B transition, as evidenced by the reappearance of B-DNA CD signals (Figure 16c). Among the anionic porphyrins, NiTPPS displayed a negative bisignate CD signal (Figure 16d), whereas H_2_TPPS and ZnTPPS showed weak interactions with Z-DNA (Figure 16d). The comparison highlights the role of porphyrin charge and metal coordination in determining their binding specificity and chiroptical responses. Cationic porphyrins, particularly ZnTMPyP4, demonstrated strong affinities and induced pronounced CD signals for both B-DNA and Z-DNA, underscoring its potential as effective probe for studying DNA polymorphism and structural transitions.

**Figure 17:**
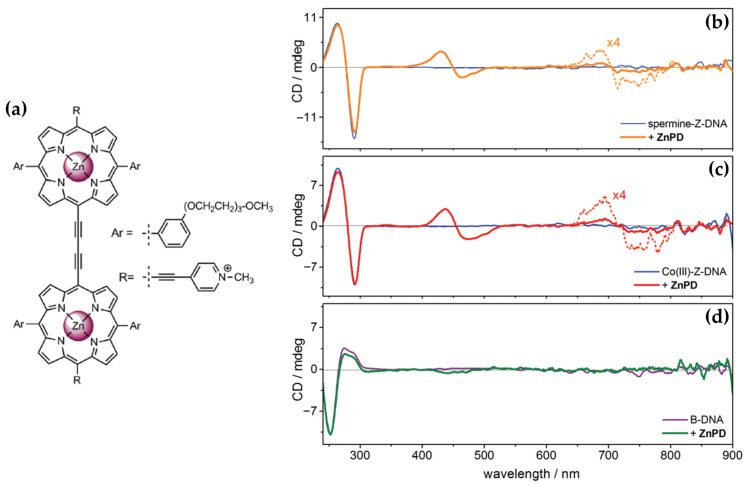
(**a**) Structure of ZnPD. CD spectra of ZnPD (5 μM) in presence of Z-form poly(dG-dC)_2_ induced by (**b**) spermine (12 μM) and (**c**) Co(NH_3_)_6_^3+^ (12 μM). (**d**) CD spectrum of ZnPD (5 μM) with B-form poly(dG-dC)_2_. Experimental conditions: [poly(dG-dC)2] = 50 μM in Na-cacodylate buffer (1 mM, pH = 7.0, 50 mM NaCl) containing 1% DMSO. Adapted with permission from ref. [99]. Copyright 2003 Royal Society of Chemistry.

Building on their previous findings, the same authors [99] later described the chiroptical properties of a conjugated zinc porphyrin dimer (ZnPD) (Figure 17a) in its interactions with both B-DNA and Z-DNA, providing insights into its specificity for different DNA conformations. CD spectra revealed that ZnPD exhibited weak interactions with B-DNA, characterized by a weak negative Cotton effect in the Soret and Q-band region (Figure 17d) at higher ZnPD-to-DNA ratios. The lack of significant CD intensity indicated minimal chiral interactions and non-disruptive external binding to the right-handed helical structure of B-DNA. In contrast, ZnPD generated strong bisignate CD signals in both the Soret and Q-band regions (Figure 17b,c) when interacting with Z-DNA, indicative of robust chiral stacking interactions with the left-handed Z-DNA helix. The CD signals were more pronounced in spermine-induced Z-DNA (Figure 17b) compared to cobalt-induced Z-DNA (Figure 17c), reflecting the ZnPD selective affinity for specific Z-DNA forms. Additionally, the intensity of the CD signals increased with higher ligand-to-DNA ratios, consistent with enhanced porphyrin aggregation along the Z-DNA helix. ZnPD also demonstrated enhanced photostability in the presence of Z-DNA, with spermine-induced Z-DNA offering the highest protection against photodegradation.

A comparison with previous works further underscores the critical role of the zinc ion in these interactions. Earlier studies revealed ZnTMPyP4 exhibited selective groove binding and strong bisignate CD signals when interacting with Z-DNA, while demonstrating weaker interactions with B-DNA. This behavior strongly suggests that the Zn(II) center plays a pivotal role in stabilizing and interacting with the chiral environment of Z-DNA through stacking and groove binding modes. The findings emphasize Zn(II)-based porphyrins as highly effective chiroptical probes for left-handed DNA conformations and valuable tools for studying DNA polymorphism and structural dynamics.

**Figure 18:**
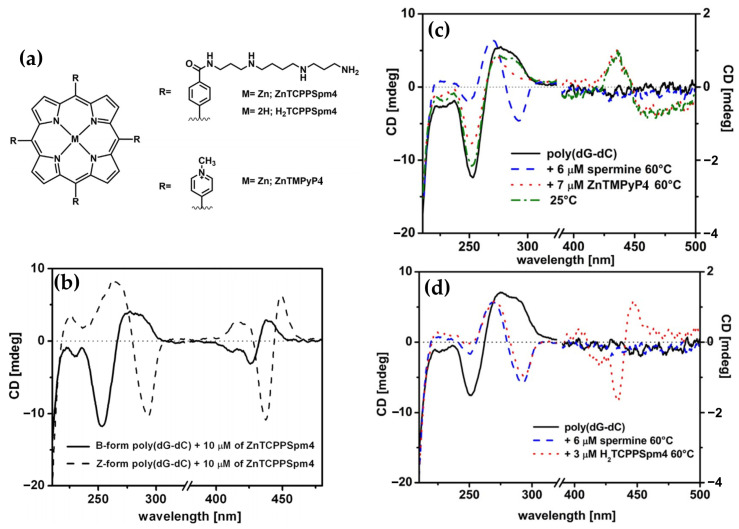
(**a**) Structures of MTCPPSpm4 and ZnTMPyP4. (**b**) CD spectra of ZnTCPPSpm4 (10 μM) in sodium cacodylate buffer (5 mM) with 10 mM NaCl at pH = 6.8 and 25 °C, recorded in presence of poly(dG-dC) (35 μM) in its B-form (solid line) and Z-form (dashed line), the latter induced by 12 μM of spermine. CD spectra of poly(dG-dC) in B-form (35 μM) in sodium cacodylate buffer (5 mM) with 10 mM NaCl at pH = 6.8, represented by the black solid line. Spectra following the addition of 6 μM spermine are shown as a blue dashed line, while the red dotted line corresponds to the addition of (**c**) 7 μM of ZnTMPyP4 at 60 °C and (**d**) 3 μM of H_2_TCPPSpm4. The green dashed-dotted line represents spectra after cooling the sample to 25 °C. Adapted with permission from ref. [100]. Copyright 2017 Elsevier.

In 2017, Gangemi et al. [100] designed the meso-tetrakis-(4-carboxysperminephenyl) porphyrin (ZnTCPPSpm4) (Figure 18a), a zinc(II)-based porphyrin derivative functionalized with four spermine groups, as a versatile molecular probe for the detection, induction, and stabilization of Z-DNA. ZnTCPPSpm4 demonstrated distinct CD signals for B-DNA and Z-DNA, enabling clear differentiation between the two conformations. The CD spectrum for B-DNA showed a positive/negative exciton split band in the Soret region (Figure 18b, solid line), indicating porphyrin–DNA interactions. In contrast, for Z-DNA it exhibited a trisignate CD band in the porphyrin absorption region (Figure 18b, dashed line), highlighting the role of ZnTCPPSpm4 as a chiroptical probe for the left-handed conformation. Although ZnTCPPSpm4 could not independently induce the B–Z transition at micromolar concentrations, it acted synergistically with subthreshold levels of spermine to catalyze the transition, producing strong Z-DNA CD signals. Notably, ZnTCPPSpm4 also stabilized the Z-DNA conformation, preventing reversion to the B-form at room temperature for up to one week, unlike spermine-induced Z-DNA, which rapidly reverted under the same conditions. This stabilization was attributed to the cooperative action of the Zn(II) center, which enabled axial coordination with guanine N7 atoms, and the spermine groups, which promoted and locked the Z-DNA structure. Comparisons with other porphyrins, such as ZnTMPyP4 (Figure 18c) and H_2_TCPPSpm4 (Figure 18d), highlighted the unique synergy of Zn(II) and spermine groups in ZnTCPPSpm4, as neither alternative could effectively stabilize Z-DNA. These findings establish ZnTCPPSpm4 as a pioneering molecular tool for probing, inducing, and stabilizing Z-DNA.

**Figure 19:**
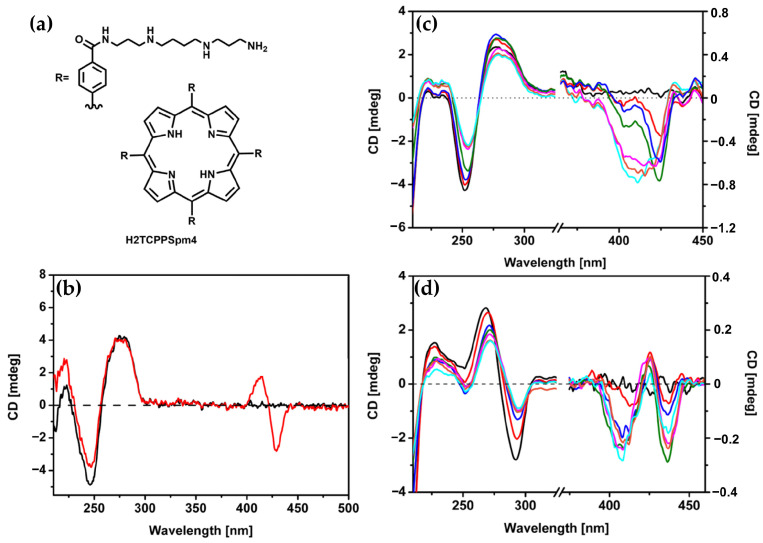
(**a**) Structure of H_2_TCPPSpm4 porphyrin. (**b**) CD spectra of ct-DNA (25 μM) in 1 mM sodium cacodylate buffer with 10 mM NaCl at pH 6.8, recorded before (black line) and after addition of 3 μM H_2_TCPPSpm4 (red line). (**c**) CD spectra of B-form poly(dG-dC)_2_ (25 μM) under the same buffer conditions (black line), with increasing concentrations of H_2_TCPPSpm4: 1 μM (red line), 2 μM (blue line), 3 μM (green line), 4 μM (pink line), 5 μM (orange line), and 6 μM (cyan line). (**d**) CD spectra of Z-form poly(dG-dC)_2_ (25 μM) induced by 9 μM of spermine in the same buffer (black line), with increasing concentrations of H2TCPPSpm4: 1 μM (red line), 2 μM (blue line), 3 μM (green line), 4 μM (pink line), 5 μM (orange line), and 6 μM (cyan line). Adapted with permission from ref. [39]. Copyright 2018 World Scientific Publishing.

Building on earlier studies exploring porphyrin derivatives bearing spermine arms and their interactions with DNA, the same authors in 2018 [39] further investigated the H_2_TCPPSpm4 porphyrin (Figure 19a), focusing on its capacity to differentiate and stabilize various DNA conformations. With ct-DNA, H_2_TCPPSpm4 exhibited bisignate induced CD signals with a negative Cotton effect at 430 nm and a positive one at 410 nm (Figure 19b). Unlike ZnTCPPSpm4, which primarily relied on groove binding, H_2_TCPPSpm4 demonstrated enhanced aggregation capabilities, driven by the spermine arms facilitating electrostatic interactions. With B-DNA in the form of poly(dG-dC)_2_, H_2_TCPPSpm4 showed a dual binding mode: slight intercalation in the end parts of the B-poly(dG-dC) sequence (end-stacking) at low porphyrin concentrations, evidenced by a negative induced CD signal in the Soret region (Figure 19c), and deeper intercalation between GC planes at higher concentrations, leading to intensified chiroptical responses. Interactions with Z-DNA (spermine-induced poly(dG-dC)_2_) revealed a trisignate induced CD signals in the Soret region (Figure 19d), which suggests the presence of intimate contact between porphyrin molecules, alongside reduced CD intensity at 265 nm and 290 nm, suggested modifications in the Z-DNA structure at higher porphyrin concentrations. Overall, H_2_TCPPSpm4 demonstrated selective binding modes across DNA conformations, with edge-to-edge aggregation along the ct-DNA helix, end-stacking or intercalative binding with B-poly(dG-dC)_2_ and face-to-face stacking with strong π–π interactions between porphyrins in the presence of Z-DNA.

**Figure 20:**
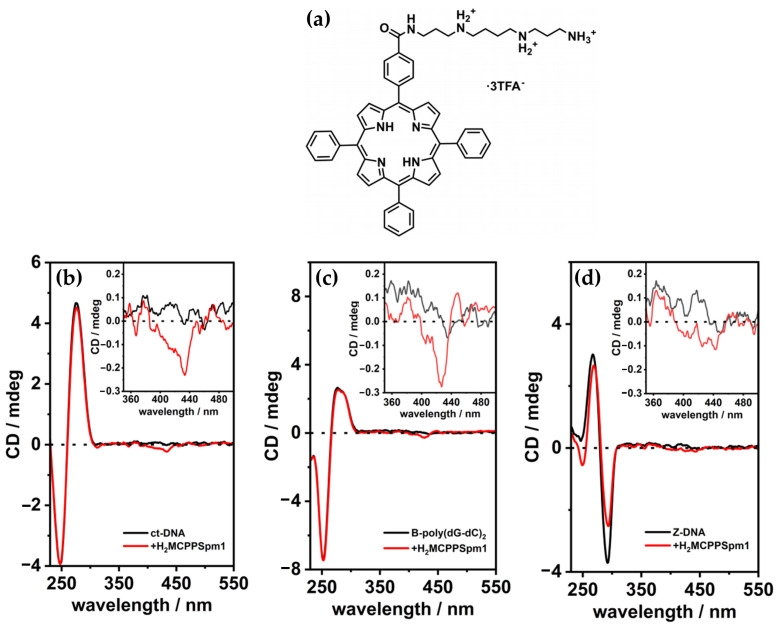
(**a**) Structure of H_2_MCPPSpm1 porphyrin. CD spectra of (**b**) ct-DNA (35 μM), (**c**) B-poly (dG-dC)_2_ (35 μM), and (**d**) Z-poly (dGdC)2 (35 μM) in cacodylate buffer 5 mM, with 10 mM NaCl at pH = 6.8, recorded before (black line) and after addition of 5 μM H_2_MCPPSpm1 (red line). Adapted with permission from ref. [101]. Published by John Wiley and Sons, 2020.

A similar study was conducted by the same group in 2020 [101], which focused on a monospermine porphyrin derivative (H_2_MCPPSpm1) (Figure 20a), instead of a tetra-spermine porphyrin, to investigate its interactions with various DNA conformations. H_2_MCPPSpm1 exhibited a distinct binding mode with ct-DNA and B-form poly(dG-dC), characterized by external stacking interactions along the DNA helix. In both cases, the porphyrin produced a negative induced circular dichroism (ICD) signal in the Soret region, centered around 430 nm (Figure 20b,c). While negative ICD signals are often indicative of intercalative binding, the observed interactions were attributed to edge-to-edge stacking of porphyrins on the DNA surface rather than intercalation. This conclusion was supported by the absence of structural distortions of DNA structure and by other spectroscopic techniques.

On the contrary, with Z-form poly(dG-dC), induced by spermine, H_2_MCPPSpm1 demonstrated a different interaction. The negative ICD signal in the Soret region (Figure 20d) suggested some intercalation of the porphyrin among the DNA bases. This was further supported by a decrease in the CD intensity of the DNA bands at 265 nm and 290 nm upon porphyrin addition, which aligns with previous findings for similar aromatic spermine derivatives. These observations indicate that monomeric porphyrins, released from aggregates at low concentrations, intercalate within the Z-DNA structure. However, the presence of spermine used to induce the B to Z transition, likely partially shields the negatively charged DNA backbone, reducing the affinity of H_2_MCPPSpm1 for Z-DNA and directing the porphyrins toward the end portions of the sequence. In these regions, porphyrins appear to have higher affinity and may destabilize the Z conformation.

These findings collectively underscore the versatility and significance of spermine-functionalized porphyrins in DNA studies. Spermine arms not only enhance electrostatic interactions and facilitate binding modes specific to DNA conformations but also influence the stabilization and discrimination of left-handed Z-DNA from right-handed B-DNA. The cooperative action of spermine groups with metal centers, as seen in ZnTCPPSpm4, represents an optimal design for stabilization and selective targeting of Z-DNA.

#### 2.1.3. Porphyrin Derivatives as Probes for the A-DNA

A-DNA (Figure 2) is a right-handed structure which can be formed from the B-DNA under specific environmental conditions such as low hydration, high ionic strength, or the presence of organic solvents. DNA–RNA hybrids and certain GC-rich sequences also favor the A-DNA form, showcasing its versatility in structural transitions [102,103,104,105,106]. This alternative form is more condensed and shorter than the typical B-form. The A-DNA consisting of more than 11 base pairs per turn, which is a larger number than the approximately 10 found in B-DNA. This results in a shorter distance between each base pair, leading to the more compact overall structure. A unique feature of the A-form is its central axial hole, which is not seen in B-DNA (Figure 2). In terms of orientation, the base pairs are inclined with respect to the helical axis, differing from their perpendicular alignment in the B-form. A further distinction of A-DNA lies in the characteristics of its grooves: the major groove is deep and narrow, while the minor groove is wide and shallow [47,48].

A-DNA is essential for cellular defense and biological processes. It protects DNA in Bacillus subtilis spores and extremophiles like the SIRV2 virus under harsh conditions [107,108,109]. The reversible B→A transition during desiccation also highlights its adaptability [110].

A-DNA facilitates protein–DNA interactions by exposing the sugar–phosphate backbone during local B→A transitions, enabling transcription and enzymatic activities [111]. Additionally, RNA and RNA–DNA hybrids frequently adopt A-DNA-like conformations, crucial for replication and transcription, while modified nucleotides mimic the A-form to enhance binding and enzymatic resistance in therapeutic applications [112,113].

In the last decade, research on porphyrin interactions with A-DNA has been surprisingly scarce, with only two significant studies published on the topic. This limited attention is striking given the unique structural and biological relevance of A-DNA, particularly its role in cellular defense mechanisms and protein–DNA interactions. While extensive investigations have been conducted on B- and Z-DNA and their interactions with porphyrins, the lack of comparable research on A-DNA highlights a gap in our understanding of how this compact and stable DNA form interacts with porphyrin derivatives. The following section examines the findings from the two studies that have explored A-DNA–porphyrin interactions, focusing on their structural and chiroptical properties

**Figure 21:**
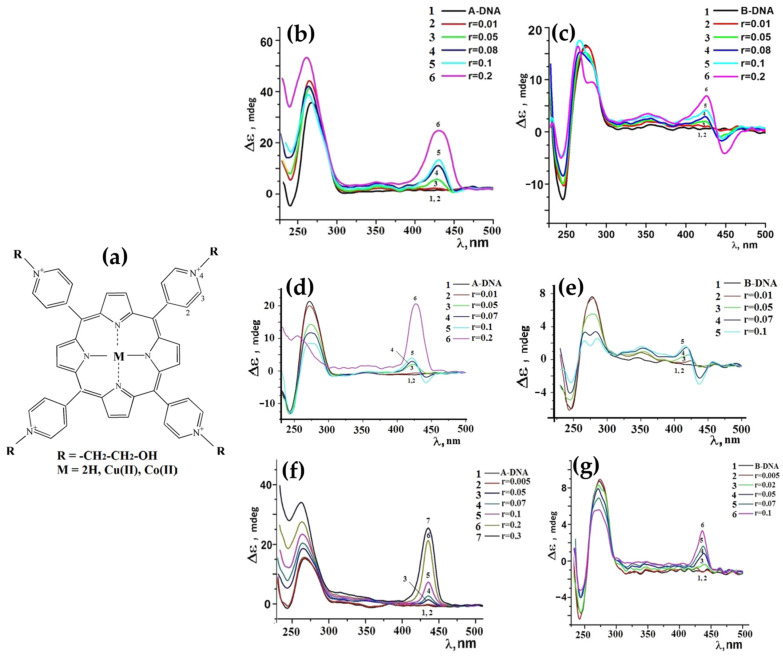
(**a**) Chemical structures of meso-tetra-(4N-oxyethylpyridyl) porphyrin (TOEPyP4) and its metallated derivatives, CuTOEPyP4 and CoTOEPyP4. Induced circular dichroism (ICD) spectra of TOEPyP4 bound to (**b**) A-DNA and (**c**) B-DNA, [DNA] = 7.5 × 10^−^^5^ M bp/mL. ICD spectra of CuTOEPyP4 bound to (**d**) A-DNA and (**e**) B-DNA, [DNA] = 4.3 × 10^−^^5^ M bp/mL. ICD spectra of CoTOEPyP4 bound to (**f**) A-DNA and (**g**) B-DNA, [DNA] = 3.2 × 10^−^^5^ M bp/mL. Each experiment was conducted at an ionic strength of [Na+] = 0.001 M, 25 °C, and pH = 7.1. Relative porphyrin-to-DNA ratio (r) is indicated in each spectrum. Adapted with permission from ref. [114]. Copyright 2017 World Scientific Publishing.

In 2017, Avetisyan and colleagues [114] explored the interaction of water-soluble meso-tetra-(4N-oxyethylpyridyl) porphyrin (TOEPyP4) and its metallated derivatives, CuTOEPyP4 and CoTOEPyP4 (Figure 21a), with both A- and B-form DNA at low ionic strength (1 mM NaCl). Using CD spectroscopy, they demonstrated that TOEPyP4 and CuTOEPyP4 exhibited stronger binding affinities for A-DNA compared to B-DNA, with binding constants approximately twice higher.

In the case of TOEPyP4, the ICD spectra of A-DNA displayed a positive band in the Soret region (Figure 21b), whose intensity increased with the porphyrin concentration, confirming the ordered external binding mode. In contrast, for B-DNA, TOEPyP4 showed bisignate ICD spectra (Figure 21c), indicative of a combination of intercalation and external binding. The presence of bisignate signals suggested the porphyrin intercalated within the DNA at lower porphyrin concentrations while shifting to external binding at higher concentrations.

For CuTOEPyP4, the interactions with A-DNA also demonstrated a positive ICD band (Figure 21d), indicating a preferential external binding mode at higher concentrations. However, at lower porphyrin-to-DNA ratios, slight intercalation was observed. The interactions with B-DNA revealed bisignate ICD spectra (Figure 21e), confirming intercalation as the dominant binding mode across all concentrations.

CoTOEPyP4, distinguished by its axial ligand, displayed a consistent external binding mode for both A- and B-DNA. The ICD spectra of CoTOEPyP4 complexes with both DNA forms were characterized by a single positive band (Figure 21f,g), reinforcing its inability to intercalate due to steric hindrance.

The thermodynamic analysis further supported the spectroscopic findings. For TOEPyP4 and CuTOEPyP4, binding with B-DNA was associated with enthalpic contributions indicative of intercalation, while interactions with A-DNA were predominantly entropic, highlighting the role of hydration effects and the release of water molecules from the DNA surface. CoTOEPyP4 interactions with both DNA forms were entirely entropic, consistent with its external binding mechanism.

This study underscored the significant differences in porphyrin binding mechanisms depending on the DNA conformation, with A-DNA showing a stronger preference for external binding, potentially due to its distinct hydration properties and structural characteristics.

**Figure 22:**
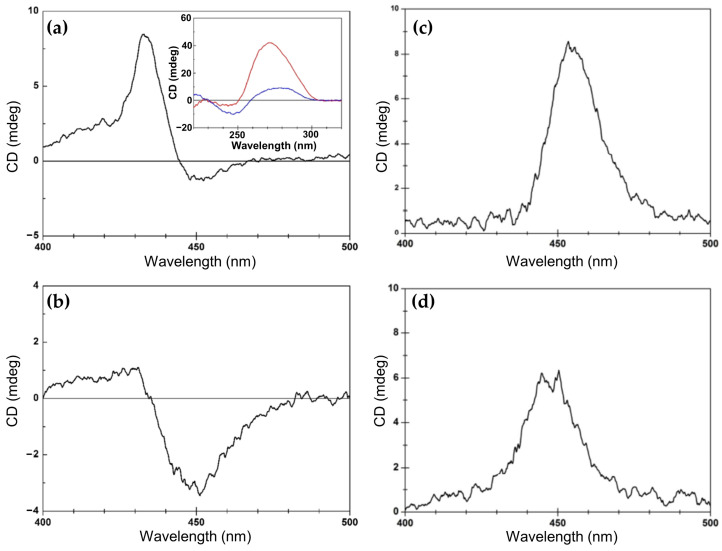
CD spectra of H_2_TMPyP4 bound to DNA in (**a**) an 80% ethanol solution and (**b**) an aqueous buffer. Inset shows CD spectra of B-DNA (blue) and A-DNA (red) in the DNA region. CD spectra of CoTMPyP4 bound to DNA in (**c**) an 80% ethanol solution and (**d**) an aqueous buffer. Experimental conditions: [H_2_TMPyP4] = 5 μM and [DNA] = 100 μM. Adapted from ref. [115]. Published by American Chemical Society, 2018; further permissions related to material excerpted should be directed to the ACS.

In 2018, Sol Oh et al. [115] investigated the interactions of H_2_TMPyP4 and its cobalt derivative (CoTMPyP4) with A- and B-DNA using CD spectroscopy. For B-DNA, H_2_TMPyP4 exhibited a negative CD signal in the Soret region under aqueous conditions (Figure 22b), indicative of an intercalative binding mode driven by π–π stacking interactions with DNA bases. Conversely, CoTMPyP4 showed a positive CD signal in the same region (Figure 22d), reflecting an external groove binding mode with self-stacking interactions along the DNA helix.

In the case of A-DNA, under 80% ethanol conditions, H_2_TMPyP4 displayed a bisignate CD pattern with a positive band at 433 nm and a negative band at 451 nm (Figure 22a), indicating an external binding mode. Similarly, CoTMPyP4 exhibited a positive CD signal (Figure 22c) consistent with groove binding. The distinct structural features of A-DNA, such as its tilted bases and shallow grooves, restricted intercalation, favoring external binding for both porphyrins.

The comparative analysis highlighted the influence of DNA conformation and environmental conditions on porphyrin binding modes. Aqueous conditions facilitated H_2_TMPyP4 intercalation with B-DNA, while ethanol-rich environments promoted external binding to A-DNA.

#### 2.1.4. Chiroptical and Structural Insights into Porphyrin Interactions with Single-Stranded DNA

Single-stranded DNA (ssDNA) plays a critical role as a transient intermediate in key genome maintenance processes, including DNA replication, repair, and recombination. Unlike its double-stranded counterpart (dsDNA), ssDNA exhibits remarkable flexibility due to the lack of a stabilizing complementary strand and typically does not form well-defined secondary structures on its own [116]. Its structure is often modeled using freely jointed or worm-like chain concepts, with a persistence length significantly shorter than dsDNA [117,118].

This flexibility allows ssDNA to accommodate numerous protein interactions crucial for genomic integrity. Proteins such as single-stranded DNA binding proteins (SSBs) are rapidly recruited to protect ssDNA from degradation and facilitate the recruitment of other DNA-processing proteins [119,120]. Additionally, helicases and recombinases interact with ssDNA to unwind dsDNA or mediate strand exchange during homologous recombination [121].

The unique properties of ssDNA and its interactions with proteins underline its central role in maintaining cellular genomic stability. Since ssDNA and proteins that bind to it both absorb strongly in the UV region, this spectral overlap complicates detailed spectroscopical studies of their interactions. Porphyrins, by ICD signals in the visible region, could provide a powerful solution to this challenge. These visible-range chiroptical signals enable precise monitoring of ssDNA-protein interactions and ssDNA conformational dynamics, offering a valuable tool for elucidating structural and functional properties with minimal spectral interference.

**Figure 23:**
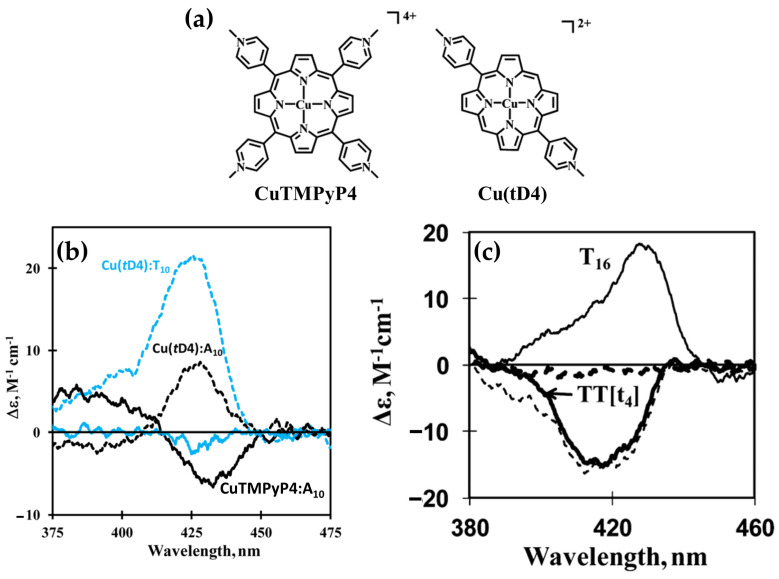
(**a**) Chemical structures of [5,10,15,20-tetra(N-methylpyridinium-4-yl)porphyrin]copper(II) CuTMPyP4 and [5,15-di(N-methylpyridinium-4-yl)porphyrin]copper(II) Cu(tD4). (**b**) Induced circular dichroism (ICD) spectra of 3.0 μM CuTMPyP4 in the presence of T10 (blue solid line) and A10 (black solid line), with strand concentrations at 24 μM. ICD spectra of 2.5 μM Cu(tD4) under similar conditions are shown for T10 (blue dashed line) and A10 (black dashed line) at 24 μM strand concentration. (**c**) ICD spectra from a competitive binding study involving Cu(tD4) interacting with excess ssDNA T16 (thin solid line), dsDNA hairpin TT[t4] (thick solid line), a mixture of both hosts (thin dashed line), and a control solution containing only the porphyrin (thick dashed line). Adapted with permission from ref. [122]. Copyright 2014, American Chemical Society.

In 2014, Gaier et al. [122] investigated the interactions of two cationic copper porphyrins, CuTMPyP4 and Cu(tD4) (Figure 23a), with single-stranded (ssDNA) and double-stranded (dsDNA) hairpin structures, employing CD spectroscopy to elucidate their binding modes. These studies revealed distinct differences in porphyrin behavior, largely dictated by their structural features and the DNA host’s architecture.

Regarding the interactions with ssDNA, CuTMPyP4, characterized by its bulky substituents, demonstrated weak interactions with ssDNA, as reflected by minimal ICD signals in the Soret region (Figure 23b, black and blue solid lines). This suggests an external binding mode with limited stacking interactions. In contrast, Cu(tD4), with reduced steric hindrance, exhibited strong positive ICD signals (Figure 23b, black and blue dashed lines), indicating pseudo-intercalation or stacking interactions with nucleotide bases. The ICD signals were particularly pronounced for pyrimidine-rich sequences, such as T10 and C10, where Cu(tD4) engaged in top-and-bottom stacking with bases. These findings highlight the adaptability of Cu(tD4) to the flexible ssDNA structure, enabling closer molecular interactions.

The study also included competitive binding experiments involving a mixture of ssDNA (e.g., T16) and a dsDNA hairpin (e.g., TT[t4]). The hairpin’s dsDNA stem, with its stable base-paired structure, served as an effective platform for porphyrin binding. Cu(tD4) and CuTMPyP4 consistently exhibit a stronger affinity for binding to dsDNA compared to ssDNA. This preference is likely due to the greater availability of bases and phosphate groups in dsDNA at the binding site, providing more interaction opportunities. ICD competitive spectra (Figure 23c) with Cu(tD4) revealed distinct binding modes: a negative ICD (Figure 23c, thick solid line) suggesting a pseudo-intercalation within the dsDNA stem and π–π stacking interactions in the single-stranded loop region. The positive ICD signals in the Soret region (Figure 23c, thin solid line) for the ssDNA bound to Cu(tD4) highlight its ability to adapt to the structural nuances of both ssDNA and dsDNA; however, the negative ICD in the mixture of both hosts (Figure 23c, thin dashed line), demonstrates a clear preference for dsDNA.

**Figure 24:**
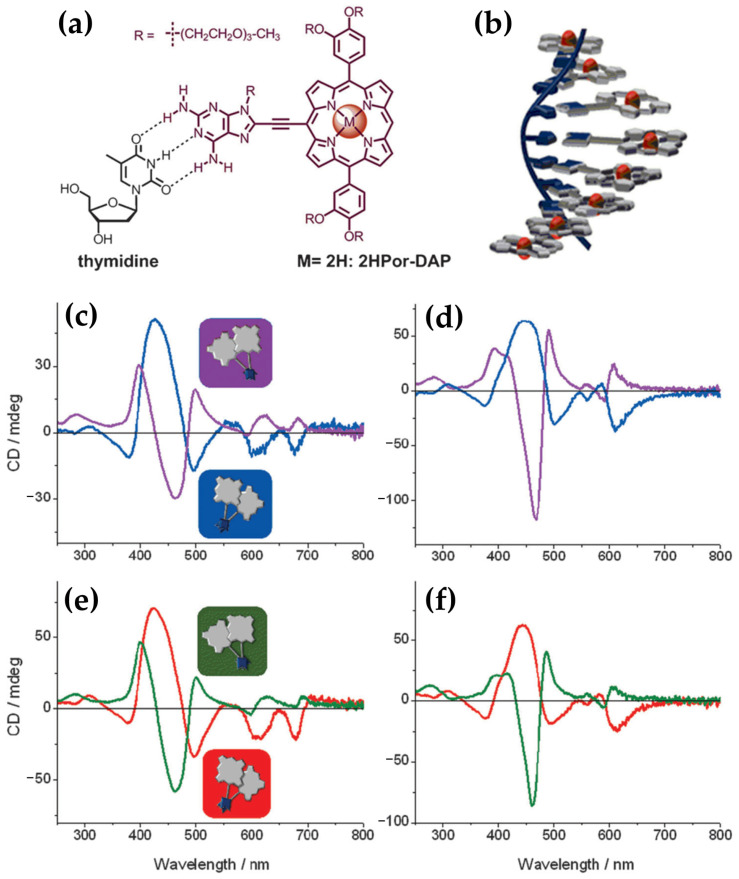
(**a**) Hydrogen bonding between porphyrin–diaminopurine conjugate 2HPor-DAP and thymidine. (**b**) Schematic representation of a right-handed DNA-templated multiporphyrin nanoassembly. CD spectra comparison of right-handed (blue and red) and left-handed (purple and green) nanoassemblies of (**c**) 2HPor-DAP:dT16, (**d**) NiPor-DAP:dT16, (**e**) 2HPor-DAP:dT40, and (**f**) NiPor-DAP:dT40. Nanoassemblies were prepared via slow annealing (10 μM 2HPor-DAP with 10 μM dT16 in 40% DMSO, Na-cacodylate buffer, 1 mM, pH = 7.0, 200 mM NaCl) or fast annealing (10 μM 2HPor-DAP with 10 μM dT16 in 45% DMSO, Na-cacodylate buffer, 1 mM, pH = 7.0, 100 mM NaCl). For NiPor-DAP:dTx assemblies, both slow and fast annealing were conducted in 40% DMSO, Na-cacodylate buffer, 1 mM, pH = 7.0, 100 mM NaCl. Adapted with permission from ref. [123]. Published by John Wiley and Sons, 2014.

In the same year, porphyrin–diaminopurine conjugates, including free-base (2HPor-DAP), nickel (NiPor-DAP), and zinc (ZnPor-DAP) derivatives (Figure 24a), were explored by Sargsyan and colleagues [123], for their ability to assemble into helical nanoarrays using single-stranded oligothymidine templates (dT8, dT16, dT40). The supramolecular structures formed through hydrogen bonding and π–π stacking interactions (Figure 24b) exhibited distinct ICD signals, demonstrating their chiroptical properties.

The helicity of the nanoassemblies was shown to be influenced by the annealing rate. Slow annealing preferentially yielded right-handed assemblies (P-helix), while fast annealing favored left-handed assemblies (M-helix) when templated by dT40 and dT16 oligothymidine. Notably, the M-isomers and P-isomers of the 2HPor-DAP:dT40 (Figure 24e) and 2HPor-DAP:dT16 (Figure 24c) nanoassemblies, as well as the M-isomers of the NiPor-DAP:dT40 (Figure 24f) and NiPor-DAP:dT16 (Figure 24d) nanoassemblies, exhibited similar CD signatures within each pair, differing only in intensity. In contrast, the nanoassemblies formed along the shortest template, dT8, produced weaker CD signals with slightly altered profiles, suggesting that the dT8 template imposes structural constraints that limit access to both helicities.

These results highlight the role of the template length and annealing conditions in controlling the chiroptical properties and helicity of the nanoassemblies. Interestingly, ZnPor-DAP failed to assemble into chiral nanostructures under the same conditions, likely due to steric hindrance from its axial water ligands.

This study underscores the potential of ssDNA-templated porphyrin assemblies for forming stable, modular, and tunable helical architectures. The ability to manipulate helicity through annealing conditions and template length provides a valuable tool for applications in biomolecular sensing, nanomaterials design, and chiral supramolecular chemistry.

**Figure 25:**
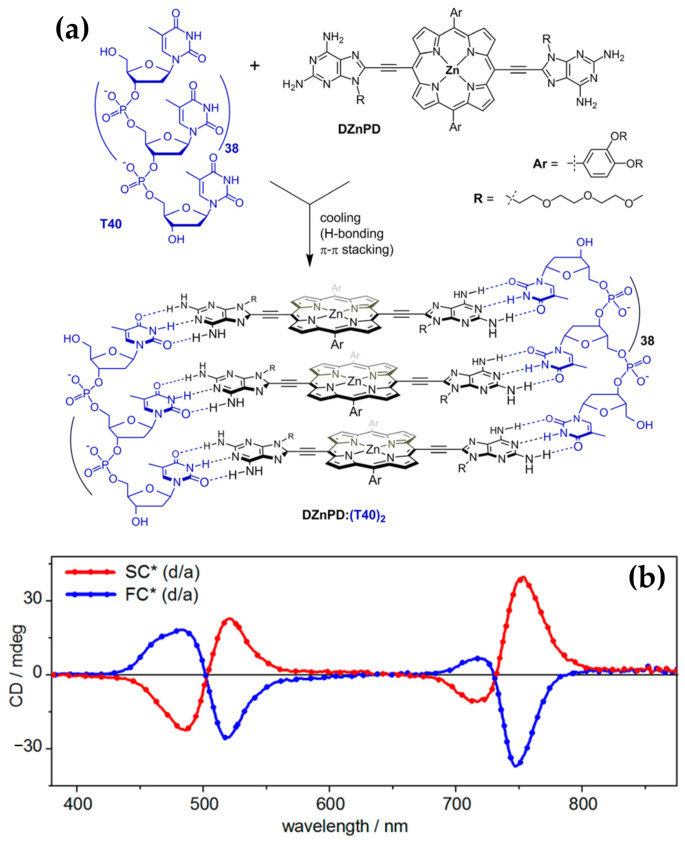
(**a**) Hydrogen bond-templated assembly of zinc(II)-porphyrin diaminopurine conjugate (DZnPD) with T40 ssDNA into chiral nanoassemblies. (**b**) CD spectra of chiral nanoassemblies following dialysis and annealing: left-handed (M)-FC*, prepared by fast cooling (blue line), and right-handed (P)-SC*, prepared by slow cooling (red line). Adapted with permission from [124]. Copyright 2020, American Chemical Society.

In 2020, the authors [124] extended previous investigations into porphyrin–DNA interactions by exploring a novel porphyrin derivative templated by single-stranded DNA (ssDNA) oligothymidylate sequences (T40) (Figure 25a). The research provided deeper insights into how DNA templates and solution conditions influence the formation and stability of chiral nanoassemblies.

Consistent with earlier findings, the helicity of the assemblies was shown to be dependent on annealing conditions: slow annealing resulted in right-handed (P) helicity with bisignate ICD signals in the Soret region (Figure 25b, red line), while fast annealing produced left-handed (M) helicity with opposite ICD spectral profiles (Figure 25b, blue line).

The newly introduced porphyrin exhibited enhanced interchromophore interactions and more robust thermal stability compared to the previously studied derivatives. Additionally, they systematically explored the influence of solution conditions, such as NaCl and DMSO concentrations, on helicity and assembly stability. For instance, assemblies formed under high NaCl concentrations (700 mM) during slow annealing displayed exceptional stability against thermal and acid–base challenges. Fast annealing under 26% DMSO, in contrast, yielded less stable but structurally distinct assemblies, underscoring the importance of solution composition in modulating assembly behavior.

Together, these studies illustrate the progression of DNA-templated chiral nanoassembly research. They emphasize the interplay between porphyrin structure, DNA templates, and solution conditions in dictating helicity, stability, and functionality.

#### 2.1.5. Chiroptical and Structural Insights into Porphyrin Interactions with G-Quadruplex DNA

G-quadruplexes (G4s) are non-canonical secondary DNA structures formed by guanine-rich sequences that naturally assemble into four-stranded configurations, a phenomenon first demonstrated in the late 1980s with DNA sequences containing runs of three or four consecutive guanines (G-tracts). These structures (Figure 2) arise from the stacking of guanine tetrads (G-quartets), which are stabilized by Hoogsteen hydrogen bonding and monovalent cations, such as potassium or sodium [125,126,127]. G-quadruplexes display significant structural diversity, adopting topologies such as parallel, antiparallel, or hybrid configurations depending on strand orientation and loop composition. They can also be classified based on their formation: intermolecular G4s, which involve two or four distinct DNA strands, and intramolecular G4s, which form from a single DNA strand [54,128].

Found abundantly in critical genomic regions, such as telomeres and promoter regions of oncogenes such as MYC and KRAS, these structures have been shown to regulate biological processes such as replication, transcription, and genome stability [129,130,131].

At telomeres, G-quadruplexes play a pivotal role in protecting chromosomal ends and regulating telomerase activity, with the latter being critical in cancer cell immortality [132]. Additionally, these structures are linked to genomic instability and are implicated in a variety of diseases, including cancer [133,134,135] and neurodegenerative disorders [136,137,138]. This biological relevance, combined with their unique structural properties, has stimulated significant interest in developing small molecules to target and stabilize G-quadruplexes for therapeutic purposes [59,139,140,141,142,143,144].

In this subsection, we will discuss the research from the past decade focused on the interactions between porphyrins and G-quadruplex DNA, with particular emphasis on the chiroptical signals observed.

**Figure 26:**
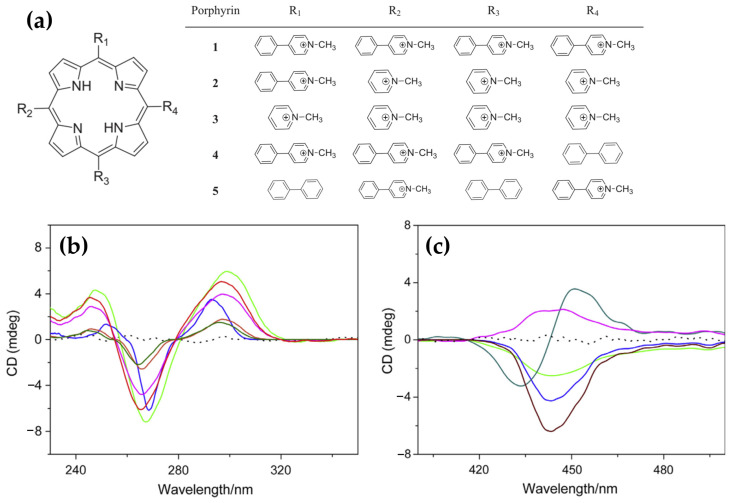
(**a**) Chemical structures of cationic porphyrins 1–5. (**b**) CD spectra in DNA region (230–350 nm) of G-quadruplex in absence (dark green line) and presence of porphyrins 1 (pink line), 2 (green line), 3 (red line), 4 (blue line), and 5 (orange line). (**c**) ICD spectra in Soret band region (390–510 nm) of G-quadruplex in presence of porphyrins 1 (green line), 2 (red line), 3 (blue line), 4 (dark green line), and 5 (pink line). Measurements were conducted in Tris buffer with a [Drug]/[DNA] ratio of 5 and a porphyrin concentration of 10 µM. Adapted with permission from ref. [145]. Copyright 2015 Elsevier.

In 2015, novel cationic expanded porphyrins (Figure 26a) were synthesized by Jin et al. [145] and their interactions with G-quadruplex DNA (G4-DNA) were investigated using spectroscopic techniques. The findings revealed distinct binding behaviors depending on the charge and structural features of the porphyrins. Tetracationic porphyrins (1, 2, and 3) displayed significant stabilization of the antiparallel G4 structure, as evidenced by pronounced increases in the positive CD band at 290 nm and the negative band at 265 nm (Figure 26b, pink, green and red lines). Conversely, tricationic and dicationic porphyrins (4 and 5) induced minimal changes in the CD spectra (Figure 26b, blue and orange lines), suggesting weaker interactions with G4-DNA.

ICD analysis further elucidated the binding modes of the porphyrins. Porphyrins 1, 2, and 3 exhibited strong negative ICD signals in the Soret band region (Figure 26c, green, red and blue lines), consistent with intercalative binding to the guanine tetrads. Porphyrin 4 (Figure 26c, dark green line) produced a bisignate ICD signal, indicative of an end-stacking mode of interaction, while porphyrin 5 showed relatively weak ICD signals (Figure 26c, pink line), pointing to weak electrostatic surface binding. These results highlight the critical role of charge density and structural design in modulating porphyrin-G4 interactions.

The study demonstrates that tetracationic porphyrins possess superior binding and stabilization capabilities for G4-DNA, underscoring their potential as therapeutic agents targeting G-quadruplexes.

**Figure 27:**
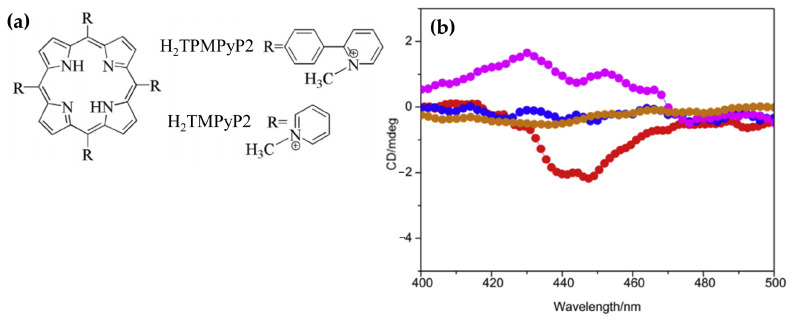
(**a**) Chemical structures of H_2_TMPyP2 and H_2_TPMPyP2 porphyrins. (**b**) ICD spectra showing G4 DNA alone (brown dotted line), H_2_TPMPyP2 alone (blue dotted line), and G4 DNA in presence of H_2_TPMPyP2 (red dotted line) and H_2_TMPyP2 (pink dotted line). Measurements were conducted in NaCl buffer with a [DNA]/[porphyrin] = 5 and a porphyrin concentration of 10 µM. Adapted with permission from ref. [146]. Copyright 2016 Elsevier.

In 2016, Zhao and coworkers [146] explored the interactions of the 5,10,15,20-tetra(phenyl-4-N-methyl-2-pyridyl) porphyrin (H_2_TPMPyP2) and 5,10,15,20-tetra(N-methyl-2-pyridyl)porphyrin (H_2_TMPyP2) (Figure 27a) with G4 DNA.

Upon binding of H_2_TPMPyP2 to AG22 G4 the CD spectra exhibited a pronounced negative ICD signal (Figure 27b, red dotted line), suggesting its ability to intercalate between G-tetrads of the quadruplex structure. This intercalative binding mode aligns with observations from fluorescence titrations and molecular docking simulations, which demonstrated favorable π–π stacking interactions between the phenyl-pyridyl moieties of H_2_TPMPyP2 and the G4 DNA. H_2_TMPyP2, in contrast, displayed weak positive ICD signals (Figure 27b, pink dotted line), indicative of external binding, likely mediated by electrostatic interactions between its cationic substituents and the DNA phosphate backbone. This external binding mode is attributed to the steric hindrance posed by the methyl groups on H_2_TMPyP2, which limit its planar alignment and intercalation into the G4 scaffold.

Additionally, the induced CD signatures corroborated the superior binding affinity of H_2_TPMPyP2, supported by its lower molecular binding energy as determined by docking studies. This affinity was reflected in the FRET melting assay, where H_2_TPMPyP2 significantly increased the melting temperature of G4 DNA compared to H_2_TMPyP2, demonstrating enhanced stabilization of the quadruplex structure. These findings underscore the critical role of structural modifications in porphyrins, such as extended planar substituents, in modulating their binding modes and affinity towards G4 DNA.

**Figure 28:**
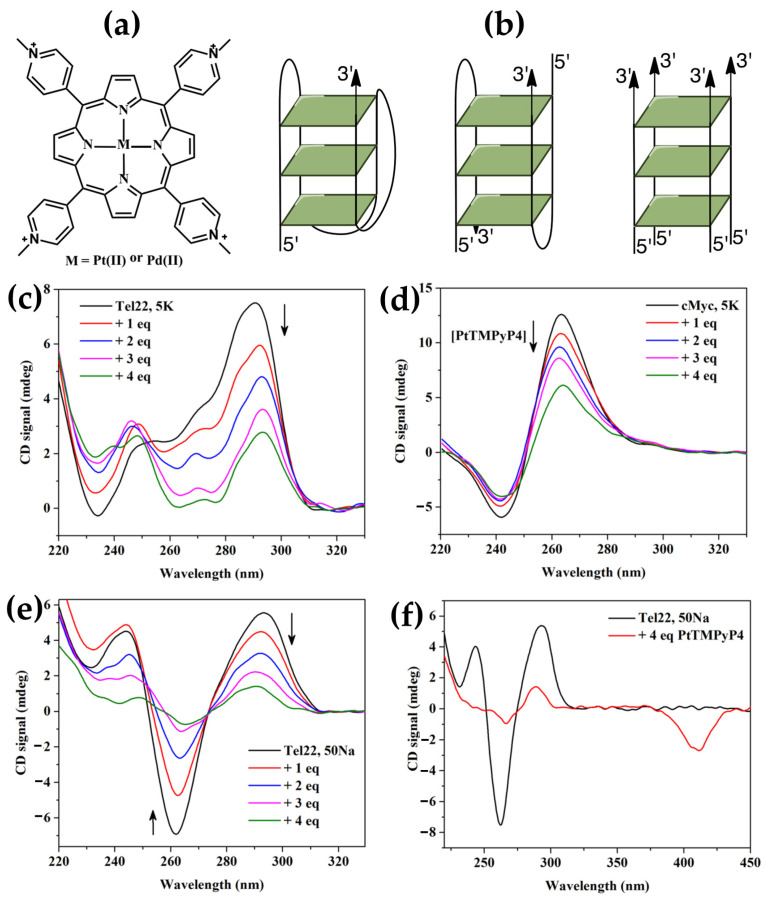
(**a**) Chemical structures of PtTMPyP4 and PdTMPyP4. (**b**) Illustration of various G-quadruplex topologies: a monomolecular mixed-hybrid GQ (left), a bimolecular antiparallel GQ (center), and a tetrastranded parallel GQ (right). PtTMPyP4 reduces the CD signal intensity of DNA quadruplexes. (**c**) CD titration of 2 µM Tel22 in 5K buffer, (**d**) CD titration of 2 µM cMyc in 5K buffer, and (**e**) CD titration of 2 µM Tel22 in 50Na buffer. (**f**) Negative induced CD signal observed at 410 nm in the Soret band region of Tel22 in 50Na buffer upon addition of four equivalents of PtTMPyP4 (corresponding to the green line in panel (**e**)). Adapted from ref. [147] under Creative Commons CC BY 4.0 license. Published by Springer Nature, 2016.

In the same year, Sabharwal et al. [147] studied the interactions of Pt(II) and Pd(II) derivatives of H_2_TMPyP4 (Figure 28a) with three representative G-quadruplex (GQ) DNA structures (Figure 28b), namely cMyc (parallel topology), Tel22 in 5K buffer (mixed-hybrid topology), and Tel22 in 50Na buffer (antiparallel topology). cMyc exhibits the typical parallel quadruplex CD signature with a peak at 265 nm and a trough at 244 nm (Figure 28d). Tel22 in 5K buffer displays a CD spectrum with peaks at 294 and 255 nm and a trough at 235 nm (Figure 28c), consistent with a mixed-hybrid structure. In 50Na buffer, Tel22 adopts an antiparallel topology, indicated by a CD spectrum with a peak at 294 nm and a trough at 260 nm (Figure 28e).

Upon the addition of PtTMPyP4, the overall CD signatures of all three GQ structures remain preserved, but a concentration-dependent decrease in CD intensity is observed (Figure 28c–e). This attenuation could be attributable to preferential porphyrin interaction with single-stranded DNA or partial DNA precipitation at high porphyrin-to-DNA ratios.

Interestingly, a negative ICD signal at approximately 410 nm, corresponding to the porphyrin’s Soret band (Figure 28f), was observed for the antiparallel Tel22 GQ upon the addition of four equivalents of PtTMPyP4. This ICD signal signifies a close π–π stacking interaction, consistent with an end-stacking binding mode. Conversely, no or very weak ICD signals were detected for cMyc or Tel22 in 5K, suggesting that their interactions with PtTMPyP4 likely occur via loops or grooves rather than direct stacking.

Authors conclude that PtTMPyP4 and PdTMPyP4 effectively bind and stabilize GQ structures, with a likely preference for end-stacking on both sides of Tel22. While both porphyrins show modest selectivity for GQ over other DNA structures, their ability to enhance quadruplex stability by over 30 °C and enhance fluorescence in the presence of quadruplexes makes them valuable tools for targeting G-quadruplex DNA in therapeutic and diagnostic applications.

**Figure 29:**
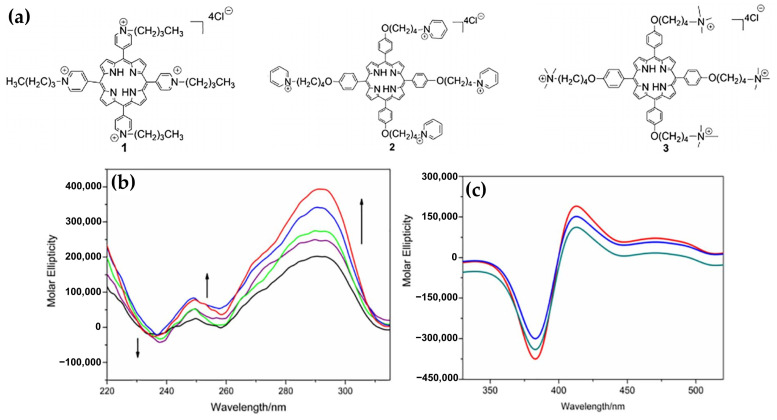
(**a**) Chemical structures of porphyrins **1**, **2**, and **3**. (**b**) CD titration spectra of 5 µM AG22 in KCl buffer, with increasing concentrations of porphyrin **3**: 0 µM (black curve), 1 µM (purple curve), 2 µM (green curve), 4 µM (blue curve), and 6 µM (red curve). (**c**) ICD spectra in KCl buffer of 1 µM porphyrins **1** (green curve), **2** (blue curve), and **3** (red curve) in presence of 5 µM G4 DNA. Adapted with permission from ref. [148]. Copyright 2017 Elsevier.

In 2017, Sun and colleagues [148] investigated the interactions of three flexible-armed cationic porphyrins with G4 DNA in mixed topology derived from human telomeres. These porphyrins, 5,10,15,20-tetra [4-(4′-pyridyl)butyloxyphenyl]porphine tetrachloride (**1**), 5,10,15,20-tetra(4-N-butylpyridyl)porphine tetrachloride (**2**), and 5,10,15,20-tetra [4-(4′-butanaminium)butyloxyphenyl]porphine tetrachloride (**3**) (Figure 29a), were specifically designed to assess how the positive charges at the ends of flexible carbon chains influenced their binding and stabilizing abilities.

The CD spectra revealed significant structural stabilization of G4 DNA upon interaction with the porphyrins, evidenced by an increase in melting temperatures (Tm). For instance, porphyrin **3** showed the highest stabilization effect (Figure 29b) compared to porphyrins **1** and **2**. The ICD signals in the Soret band region indicated mixed binding modes, including end-stacking and groove binding. Bisignate ICD signals (Figure 29c) for all porphyrin–DNA complexes confirmed this mixed interaction mode.

Furthermore, molecular docking studies suggested that the flexible arms of porphyrin **3** allowed strong electrostatic interactions with the G4 DNA backbone, along with efficient π–π stacking with the G-quartet. This unique binding geometry, attributed to the free rotation of the carbon chains, enabled porphyrin **3** to achieve higher binding affinity than porphyrins **1** and **2**.

Overall, the study highlighted the importance of the structural flexibility and positive charges of porphyrin side chains in enhancing the stabilization and folding rates of G4 DNA, making these porphyrins promising candidates for anticancer drug development due to their photodynamic properties and significant cytotoxicity against cancer cells under visible light irradiation.

**Figure 30:**
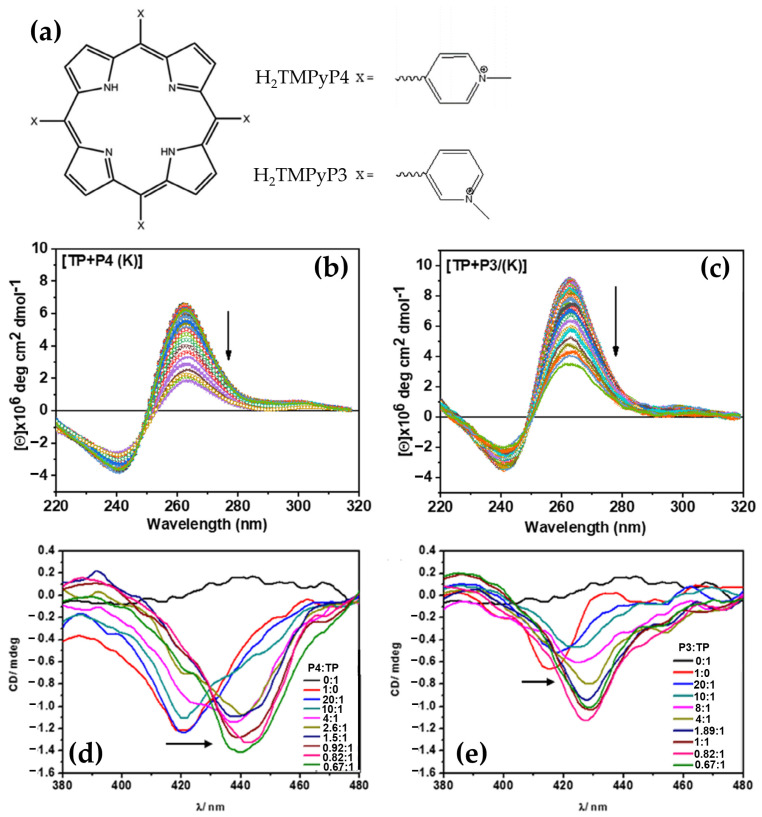
(**a**) Chemical structures of H_2_TMPyP4 and H_2_TMPyP3 porphyrins. (**b**) CD titration spectra of TP with H_2_TMPyP4 and (**c**) with H_2_TMPyP3, showing changes in the DNA region, the arrows indicate how the CD signal changes with increasing porphyrin concentration. (**d**) Induced CD spectra of H_2_TMPyP4 and (**e**) H_2_TMPyP3 as the [porphyrin]/[TP] molar ratio varies. Experiments were conducted in 20 mM sodium cacodylate buffer (pH = 7.0) containing 100 mM KCl and 0.1 mM EDTA. Adapted with permission from ref. [149]. Copyright 2022 John Wiley and Sons.

In a recent work [149] conducted by Joshi et al., the interactions of cationic porphyrin isomers H_2_TMPyP3 and H_2_TMPyP4 (Figure 30a) with G-quadruplex (GQ) DNA formed by 33-mer (TP) regulatory sequence present in the MRP1 promoter region were thoroughly examined. These porphyrins, widely recognized for their DNA binding versatility, demonstrated distinct behaviors towards GQ structures. Using CD spectroscopy, the GQ topology of TP was initially characterized, revealing the typical parallel GQ structures in the absence of porphyrins. Upon the addition of H_2_TMPyP4, significant perturbations in the GQ CD signals were observed, including reduced Cotton effects and altered peak positions in the 260–295 nm range (Figure 30b), indicating destabilization of the GQ architecture. This destabilization, attributed to the strong binding affinity of H_2_TMPyP4, was hypothesized to interfere with the base stacking and overall stability of the GQ.

In contrast, H_2_TMPyP3 showed a comparatively weaker impact on GQ stability, as evidenced by milder changes in the CD spectra (Figure 30c), suggesting less effective π–π stacking or end-stacking interactions. Notably, induced CD (ICD) signals in the Soret region provided further insights into the porphyrin binding modes. H_2_TMPyP4 displayed a pronounced negative ICD band (Figure 30d), indicative of intercalation or strong end-stacking interactions, while H_2_TMPyP3 yielded less defined ICD profiles (Figure 30e), reinforcing its weaker interaction.

These findings underscore the role of positional isomerism in modulating porphyrin–GQ interactions, with H_2_TMPyP4 emerging as a potent GQ destabilizer. The study highlights the potential of cationic porphyrins as modulators of GQ DNA structures, offering avenues for gene regulation strategies targeting the MRP1 promoter.

**Figure 31:**
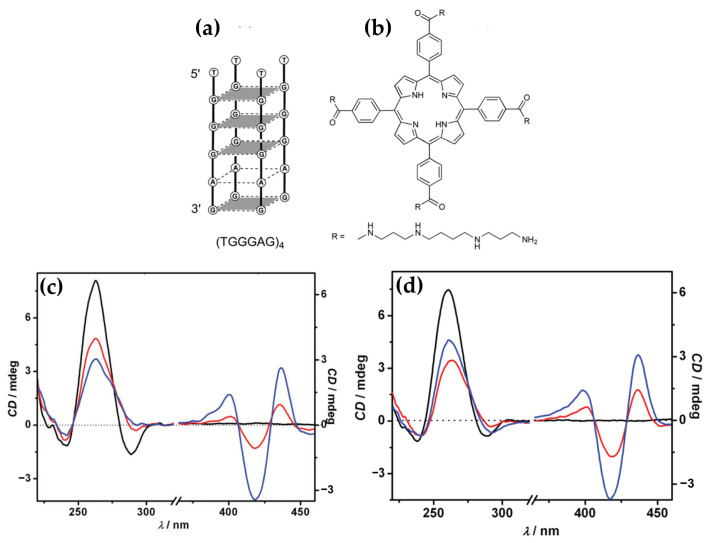
(**a**) Structure of tetramolecular G-quadruplex (GQ) formed by TGGGAG and of (**b**) porphyrin derivative H_2_TCPPSpm4. (**c**) CD spectra in K^+^ buffer of 2 µM (TGGGAG)_4_ alone (black curve) and in presence of H_2_TCPPSpm4 at different concentrations obtained through titration: 2 µM (red curve) and 4 µM (blue curve), with porphyrin added incrementally from 0.25 µM to 5.5 µM. (**d**) CD spectra in K^+^ buffer of 2 µM (TGGGAG)_4_ alone (black curve) and with H_2_TCPPSpm4 added in a single step at final concentrations of 2 µM (red curve) or 4 µM (blue curve). Adapted with permission from ref. [150]. Copyright 2017 Royal Society of Chemistry.

In 2017, D’Urso et al. [150] explored the interaction of the tetracationic porphyrin derivative H_2_TCPPSpm4, bearing spermine arms at the meso positions (Figure 31b), with the G-quadruplex (GQ) formed by the DNA aptamer TGGGAG (Figure 31a). Employing spectroscopic techniques and electrophoretic methods, the researchers demonstrated that the porphyrin’s binding and its effects on the GQ topology are dependent on the stoichiometry and the method of complex formation. At 1:1 H_2_TCPPSpm4:GQ ratio, the UV-Vis spectra displayed a notable hypochromic effect (~50%) and a redshift in the Soret band by 15 nm, indicating strong end-stacking interactions with the terminal G-tetrad. The ICD signal in the Soret region exhibited a trisignate pattern, with positive Cotton effects at 400 and 436 nm and a negative Cotton effect at 418 nm (Figure 31c), further corroborating efficient π-stacking interactions.

Interestingly, titration experiments revealed that the addition of a second equivalent of H_2_TCPPSpm4 altered the GQ conformation. CD experiments indicated partial destabilization of the GQ structure at this stoichiometry, marked by a reduced positive band at 263 nm and the near disappearance of the negative band at 290 nm (Figure 31c), characteristic of the GQ dimer (TGGGAG)_8_. This destabilization was attributed to the porphyrin’s interference with the hydrogen bonds and π-stacking within the GQ.

To further explore the influence of preparation methods, the study compared samples prepared by titration and single addition of H_2_TCPPSpm4. Interestingly, the CD spectra of the 1:1 complexes prepared by both methods (Figure 31c,d) were nearly identical in both the UV and Soret regions, indicating similar binding modes. However, significant differences were observed for the 2:1 complexes. The CD spectrum of the sample prepared by single addition of 4 µM H_2_TCPPSpm4 showed a pronounced negative Cotton effect at 290 nm (Figure 31d), indicative of a 3′-3′ stacked dimer formation, which was absent in the titration-prepared sample. Furthermore, the intensity of the positive Cotton effect at 263 nm was higher for the 2:1 sample prepared by single addition compared to the 1:1 ratio, whereas the opposite trend was observed in titration experiments.

CD melting experiments demonstrated significant differences between complexes formed via titration versus single addition. The 1:1 complex decreased the GQ’s melting temperature (Tm) by ~30 °C, highlighting its destabilizing behavior. Conversely, the single addition of two equivalents of H_2_TCPPSpm4 produced a biphasic melting curve, indicating the formation of stable GQ superstructures with an elevated Tm (~80 °C).

These findings underline the dual role of H_2_TCPPSpm4 in modulating GQ stability and topology, emphasizing the relevance of stoichiometry and preparation methods.

One year later, the H_2_TCPPSpm4 porphyrin (Figure 31b) and its Zn(II) derivative (ZnTCPPSpm4) were further investigated, focusing on their interactions with GQ DNA structures, particularly the Tel22 human telomeric GQ [40]. These porphyrins exhibited strong binding affinities to the GQ DNA, with association constants (Ka) in the range of (5–14) × 10^6^ M^−^^1^. This high binding affinity translated to a remarkable selectivity, with a ratio of 200–300 over double-stranded DNA (dsDNA). The porphyrins not only stabilized the GQ structures, but also enhanced their resistance to thermal denaturation.

**Figure 32:**
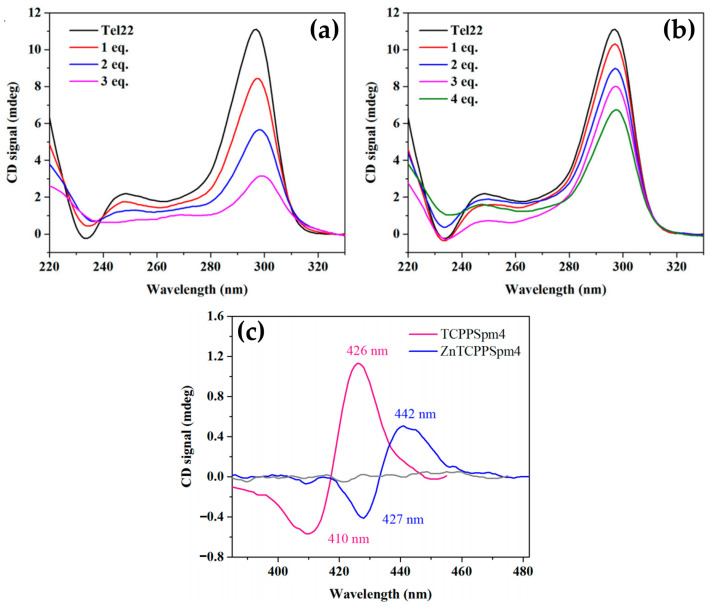
CD titration spectra of 15.0 µM Tel22 with increasing equivalents of (**a**) H_2_TCPPSpm4 and (**b**) ZnTCPPSpm4, up to four equivalents. (**c**) ICD spectra of H_2_TCPPSpm4-Tel22 and ZnTCPPSpm4-Tel22 complexes prepared at stoichiometric porphyrin-to-DNA ratios (4:1 for H_2_TCPPSpm4 and 12:1 for ZnTCPPSpm4). All data were normalized to 1 µM porphyrin concentration, with CD spectrum of porphyrin alone shown in grey. Adapted from ref. [40] under Creative Commons CC BY 4.0 license; published by MDPI AG, 2018.

CD spectroscopy revealed that the addition of H_2_TCPPSpm4 or ZnTCPPSpm4 preserved the characteristic GQ topology, as indicated by the unchanged GQ-specific CD signals. However, porphyrins lead to a dramatic decrease in the intensity of 295 nm peak (Figure 32a,b).

One proposed mechanism is the porphyrins binding to the GQ DNA by disrupting and replacing one or more G-tetrads. This disruption leads to decreased tetrad stacking, which consequently diminishes the overall CD signal. Despite this reduction in signal, the GQ structures remain stable due to the porphyrins’ stabilizing effects.

In addition, bisignate ICD signals in the Soret band region (Figure 32c) provided evidence for π–π stacking interactions between the porphyrins and the GQ ends. These ICD signals were indicative of end-stacking as the primary binding mode. Furthermore, at higher porphyrin concentrations, porphyrin self-association was observed, leading to the formation of aggregates that further stabilized the GQ structures.

Fluorescence spectroscopy corroborated these findings, highlighting the enhanced stabilization effect of ZnTCPPSpm4 compared to H_2_TCPPSpm4. This enhancement was attributed to the metal center in ZnTCPPSpm4, which likely facilitated additional interactions. UV-Vis spectroscopy also confirmed the high binding stoichiometry and provided complementary evidence of the strong porphyrin-GQ interactions.

These results underscore the effectiveness of spermine-functionalized porphyrins in targeting GQ DNA, with high specificity and strong stabilization effects. Their ability to selectively bind GQ over dsDNA, combined with their induced chiroptical signals, make these porphyrins as powerful tools for studying GQ structures and exploring their potential therapeutic applications.

**Figure 33:**
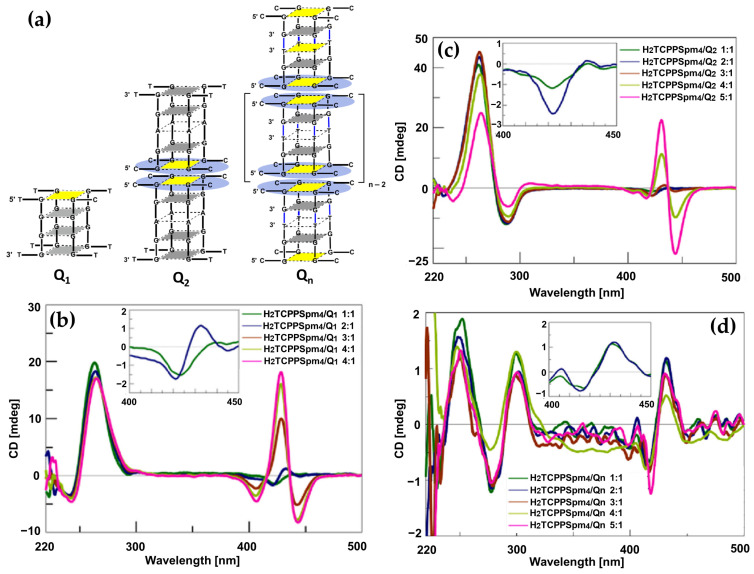
(**a**) Schematic representation of G4 models Q1, Q2, and Qn. Gray indicates G-tetrads, while G-tetrads involved in 5′-CG sticky ends are highlighted in yellow. Light blue circles represent planar octad G(:C):G(:C):G(:C):G(:C) system defining the 5′-CG sticky ends. The 3′-3′ phosphodiester bonds are marked in blue. (**b**) CD spectra of H_2_TCPPSpm4/Q1 complexes at increasing ratios (1:1 to 5:1). Inset: Comparison of H_2_TCPPSpm4/Q1 at ratios of 1:1 and 2:1. (**c**) CD spectra of H_2_TCPPSpm4/Q2 complexes at ratios from 1:1 to 5:1. Inset: H_2_TCPPSpm4/Q2 at ratios of 1:1 and 2:1. (**d**) CD spectra of H_2_TCPPSpm4/Qn complexes at ratios from 1:1 to 5:1. Inset: Comparison of H_2_TCPPSpm4/Qn at ratios of 1:1 and 2:1. Adapted from ref. [151] under Creative Commons CC BY 4.0 license; published by Elsevier, 2024.

Considering the demonstrated ability of H_2_TCPPSpm4 (Figure 31b) to bind to GQ DNA with high specificity, this porphyrin was recently explored further to investigate its interactions with higher-order GQ structures [151]. Specifically, the study examined tetramolecular G-quadruplex monomers (Q1), dimers (Q2), and extended G-wire assemblies (Qn) (Figure 33a), using advanced analytical methods such as CD, UV–Vis, fluorescence spectroscopy, PAGE, RLS, AFM, and HPLC-SEC. These experiments aimed to elucidate the binding modes of H_2_TCPPSpm4 and its potential in stabilizing and characterizing these complex GQ architectures.

In particular, for GQ monomer Q1 at lower porphyrin-to-DNA ratios (1:1 and 2:1), a negative ICD band at 425 nm (Figure 33b) suggested that H_2_TCPPSpm4 primarily end-stacked on the 5′ and 3′ faces of Q1, whereas at higher ratios (3:1 to 5:1), a trisignate ICD signal appeared, with two negative bands at 406 nm and 442 nm and a positive band at 428 nm (Figure 33a). This progression highlighted the formation of high molecular weight porphyrin–Q1 aggregates. For Q2 (Figure 33c) and Qn (Figure 33d), the porphyrin displayed a groove-binding preference, with additional stacking interactions evident at higher concentrations. CD spectra revealed distinct induced circular dichroism signals in the Soret region, varying with both the GQ structure and porphyrin concentration, reflecting the ligand’s adaptability. Moreover, AFM and PAGE analyses confirmed the formation of multimeric assemblies at higher porphyrin concentrations, particularly for G-wire structures.

#### 2.1.6. Chiroptical Signals and Interactions of Porphyrin Derivatives with I-Motif and E-Motif Structures

The i-motif is a non-canonical four-stranded DNA structure formed in cytosine-rich sequences (Figure 2). It is stabilized through hemi-protonated cytosine–cytosine+ (C:C+) base pairs that intercalate in an antiparallel orientation. This unique structure was first described by Gehring et al. [152], demonstrating its ability to form under acidic conditions. Despite initial beliefs that i-motifs are restricted to low pH environments, studies have revealed their formation under neutral pH in certain conditions [153,154], such as molecular crowding [155], negative superhelicity [61], and in the presence of specific cations [156].

Structurally, i-motifs exhibit two parallel-stranded duplexes intercalated to create a tetramolecular structure, with narrow minor grooves that contribute to its distinct topology. Their formation can be intramolecular, with folding driven by four cytosine tracts within a single strand, or intermolecular, involving multiple strands [157].

From a biological perspective, i-motifs have been recently detected in vivo [158], notably in regulatory regions of the genome, such as promoters and telomeric regions, where they are hypothesized to influence transcription and gene regulation [159,160]. Some proteins specifically interact with i-motif structures, underscoring their role as regulatory elements and potential targets for therapeutic applications [57,161,162]. Despite significant progress in i-motif structural biology, many aspects still require further study. Current data suggest that i-motifs form transiently within cells, but more in vivo studies are needed to confirm i-motif formation during various stages of the cell cycle. Additional research on how proteins and small ligands recognize i-motifs, both in vitro and in vivo, is essential to understand their roles in biological processes.

Although much focus has been placed on ligands stabilizing G-quadruplexes, fewer studies have explored their counterparts for i-motifs, partly due to the intrinsic instability of i-motifs under physiological conditions and the challenges of accessing their compact structure.

Among the few studied ligands [56,163,164], porphyrins, particularly H_2_TMPyP4, stand out as a significant class. H_2_TMPyP4, a well-known ligand for G-quadruplexes, has also been shown to interact with i-motif structures. Studies reveal that H_2_TMPyP4 binds near the ends of i-motifs via non-intercalative, electrostatic interactions without disrupting their original structure [165]. For instance, the binding of H_2_TMPyP4 to an intramolecular i-motif based on the telomeric sequence or a tetrameric i-motif formed by the A2C4 sequence demonstrated its ability to stabilize the i-motif under mildly acidic conditions. Notably, up to two H_2_TMPyP4 molecules can independently bind to a single i-motif structure, with binding constants in the micromolar range [166].

In the context of the limited studies on the interaction of porphyrins with i-motif structures, the work by Tingxiao Qin and collaborators [167] represents the only study in the literature to comprehensively investigate the ICD signal of porphyrin binding to i-motifs. This highlights the existing gap in research on porphyrin–i-motif interactions, emphasizing the need for further exploration in this promising field.

**Figure 34:**
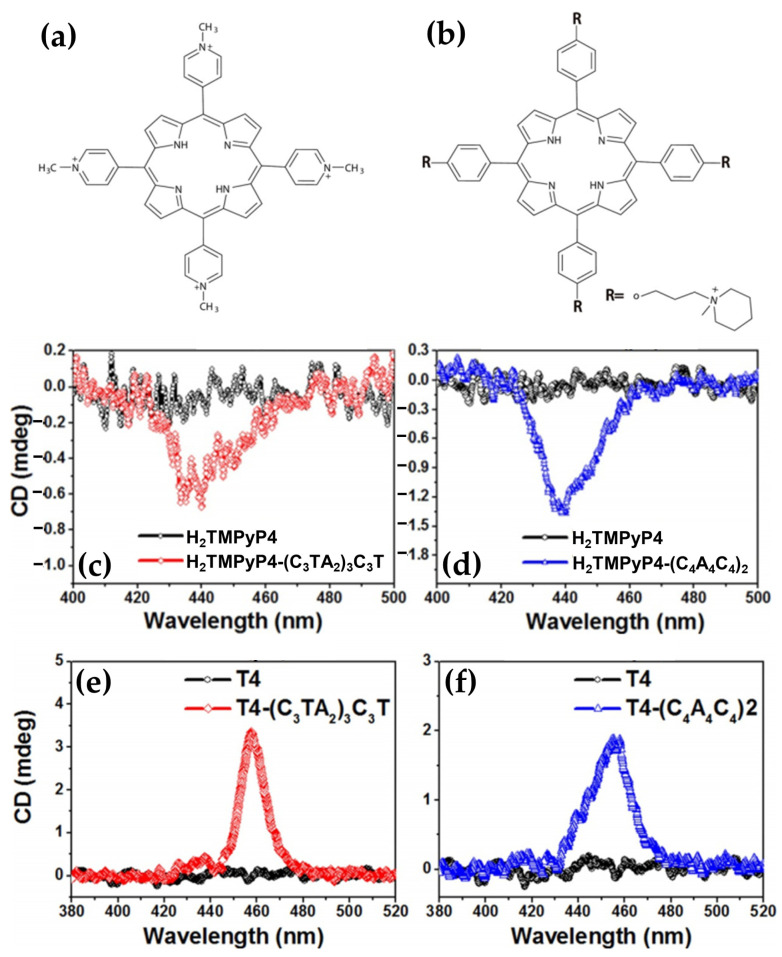
Chemical structures of (**a**) H_2_TMPyP4 and (**b**) T4 porphyrins. Induced circular dichroism (ICD) spectra of H_2_TMPyP4 (5 µM) with i-motif (30 µM) in 10 mM sodium cacodylate buffer (pH = 5.0): (**c**) (C_3_TA_2_)_3_C_3_T (red) and (**d**) (C_4_A_4_C_4_)_2_ (blue). ICD spectra of T4 (2 µM) with i-motif (12 µM) in 10 mM sodium cacodylate buffer (pH 5.0): (**e**) (C_3_TA_2_)_3_C_3_T (red) and (**f**) (C_4_A_4_C_4_)_2_ (blue). Adapted with permission from ref. [167]. Copyright 2017 John Wiley and Sons.

In particular, Qin et al. [167] revealed the binding interactions between H_2_TMPyP4 and i-motif DNA structures, highlighting the influence of ionic strength on binding modes and chiroptical properties. For the i-motif (C_3_TA_2_)_3_C_3_T, a weak negative ICD band at 440 nm (Figure 34c) was observed and a similar signal appeared at the same wavelength for (C_4_A_4_C_4_)_2_ (Figure 34d), corroborating intercalation as the dominant binding mode under these conditions.

The study further highlighted discrepancies between binding modes reported in earlier NMR experiments [165], which suggested a major groove binding mode for H_2_TMPyP4 with the (AACCCC)_4_ i-motif. This difference was attributed to variations in experimental conditions, particularly to the ionic strength. The intercalation mode, characterized by negative ICD signals, was favored in buffers with low ionic strength (10 mM sodium cacodylate). In contrast, higher ionic strengths (150 mM KCl) prevented intercalation, leading to predominant major groove binding.

The influence of ionic strength was further validated through UV-Vis and transient absorption spectroscopy. Increased ionic strength resulted in reduced intercalation, evidenced by decreased redshifts and hypochromicity in the Soret band, alongside alterations in transient absorption decay curves. A progressive increase in ionic strength shifted the binding distribution, decreasing intercalation and enhancing groove binding.

Additionally, the destabilizing effect of intercalation on the i-motif was demonstrated through melting temperature (Tm) measurements. At low ionic strength, intercalation dominated (~80%) and led to a reduction in Tm, reflecting structural destabilization. Conversely, at higher ionic strengths, where groove binding prevailed, no significant changes in Tm were observed.

Lastly, the interaction with a long-armed porphyrin derivative, 5,10,15,20-tetra-{4-[2-(1-methyl-1-piperidinyl)propoxy]phenyl}porphyrin (T4) (Figure 34b), with the methylpyridyl substituents of H_2_TMPyP4 replaced by a larger substituent was investigated. T4 exhibited positive ICD signals at 460 nm (Figure 34e) and 457 nm (Figure 34f) for (C_3_TA_2_)_3_C_3_T and (C_4_A_4_C_4_)_2_, respectively. These positive ICD bands confirmed an exclusive groove-binding mode for T4, attributed to the steric hindrance of its bulky side arms, which entirely blocked intercalative interactions.

This work underscores the complexity of H_2_TMPyP4–i-motif interactions, where binding modes and structural impacts are highly dependent on experimental conditions, making H_2_TMPyP4 a versatile tool for studying i-motif structures and their potential biological relevance. Additionally, T4 revealed an effective prohibition of intercalation into the C:C+ base pair of i-motifs, exhibiting a pure major groove binding mode. This novel property of T4 suggests the possibility of designing groove-binding ligands with site selectivity for DNA i-motifs.

In the last paper of this subsection, the focus shifts from i-motif interactions to the extrahelical conformations of DNA trinucleotide repeats, known as E-motifs. E-motifs, formed due to mismatched cytosines adopting extrahelical conformations, are prevalent in trinucleotide repeats (TRs), which can fold into homoduplexes or hairpins.

The formation of the E-motif is driven by favorable stacking interactions at pseudo-GpC steps around mismatched cytosines, aligning towards the 5′ direction within the minor groove [168]. Biologically, E-motifs are pivotal in the expansion of trinucleotide repeats, a process closely linked to various neurodegenerative disorders such as Huntington’s disease and fragile X syndrome [169,170]. These unique structural features make E-motifs promising targets for small-molecule recognition, highlighting their potential in therapeutic and diagnostic applications [171,172]. However, no ligand has yet been shown to specifically resolve the E-motif conformation within TRs.

**Figure 35:**
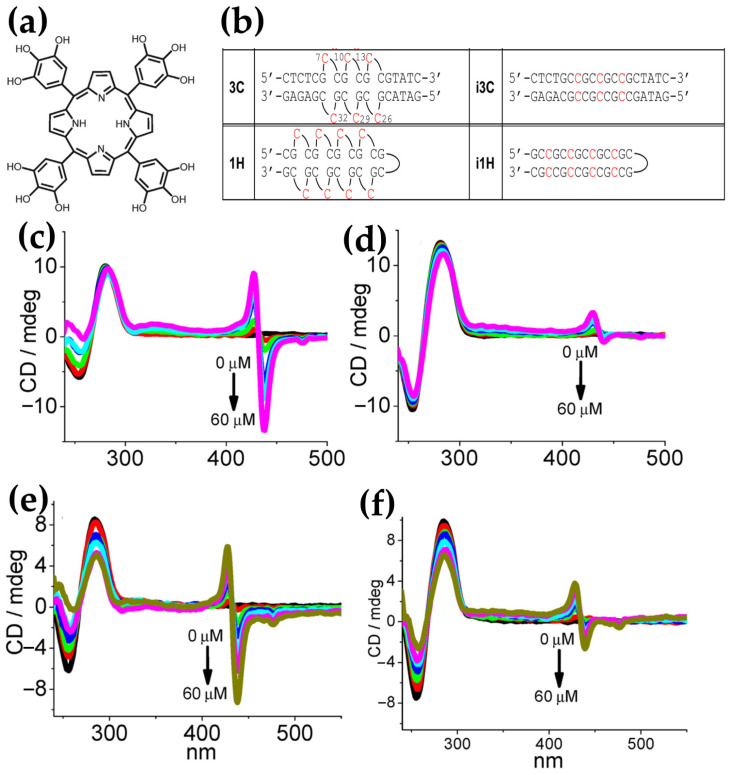
(**a**) Chemical structure of the POH3 porphyrin. (**b**) Sequences of oligonucleotides used in this study, with mismatched cytosines highlighted in red and E-motif-adopting cytosines numbered. CD spectra of (**c**) 3C and (**d**) i3C (8 μM) recorded with increasing concentrations of POH3 from 0 to 60 μM. CD spectra of (**e**) 1H and (**f**) i1H (4 μM) recorded with increasing concentrations of POH3 from 0 to 60 μM. Experiments were conducted in 10 mM PBS buffer (pH = 6.8) containing 100 mM Na^+^. Adapted with permission from ref. [173]. Copyright 2021 Elsevier.

In response to this challenge, Zhang et al. [173] synthesized a porphyrin derivative, POH3 (Figure 35a), to target E-motif cytosines through complementary hydrogen bonding. The design of POH3, featuring hydroxyl-substituted phenyl groups, exhibits high hydrophilicity and a nonplanar configuration relative to the porphyrin core, which prevents efficient intercalation with canonical duplex DNAs. This structural property minimizes nonspecific interactions, enabling selective recognition of E-motif cytosines through complementary hydrogen bonding.

CD experiments revealed that the binding is TR length- and polarity-dependent, with strong ICD signals specific to E-motif DNAs. The duplex 3C (Figure 35b), containing three E-motif cytosine–cytosine mismatches, displayed a distinct bisignate ICD signal with a positive band at 428 nm and a negative band at 438 nm (Figure 35c), indicating efficient binding of POH3 through complementary hydrogen bonding in the minor groove. This interaction slightly modified the 3C conformation, as evidenced by moderate changes in its CD signal at 254 nm (Figure 35c). In contrast, the polarity-inverted i3C duplex (Figure 35b) exhibited significantly weaker ICD signals, with a 24-fold lower intensity at 438 nm (Figure 35d), highlighting the specificity of POH3 for the E-motif conformation.

Similarly, the 1H hairpin (Figure 35b), which forms an E-motif structure in its stem, showed strong ICD responses with characteristic negative and positive bands at 438 nm and 428 nm (Figure 35e), respectively, confirming the preferential binding of POH3. The i1H hairpin, lacking the E-motif, exhibited much weaker ICD signals (Figure 35f) under identical conditions, further demonstrating the specificity of POH3. The binding stoichiometry was determined to be 2:1, with the binding primarily driven by complementary hydrogen bonding between POH3 and the E-motif cytosines.

When tested with trinucleotide repeats, POH3 exhibited strong specificity for (CCG)_9_ repeats, which form the E-motif, while showing negligible interaction with the polarity-inverted (GCC)_9_ repeats. The (CCG)_9_ repeats also significantly enhanced POH3 fluorescence, supporting its potential as a selective probe for E-motif-containing sequences.

Overall, this study underscores the ability of POH3 to selectively recognize and bind E-motif DNA via a unique hydrogen bonding mechanism, offering a promising tool for exploring trinucleotide repeat structures and their implications in neurological disorders.

### 2.2. RNA Structural Diversity

RNA is a flexible and essential biomolecule that plays different roles beyond serving as a passive carrier of genetic information. Its diverse biological functions, ranging from covering catalysis, regulation, protein synthesis, and genome maintenance, are largely dictated by its intricate structures. RNA adopts hierarchical configurations: the primary structure represents the nucleotide sequence; the secondary structure arises from canonical base-pairing (AU, CG, and GU); the tertiary structure reflects the three-dimensional folding, and the quaternary structure is the interaction with other molecules, which are often either proteins or other RNA strand interactions [174].

An important structural component distinguishing RNA from DNA is the hydroxyl group at the 2′ position of the ribose sugar, which typically imposes to the RNA helix an A-form geometry, similar to the A-DNA form. This conformation results in a deep, narrow major groove and a shallow, wide minor groove [175].

However, the regular A-form helix of RNA is often disrupted by regions of mismatched bases that allow RNA to adopt more complex secondary and three-dimensional structures.

**Figure 36:**
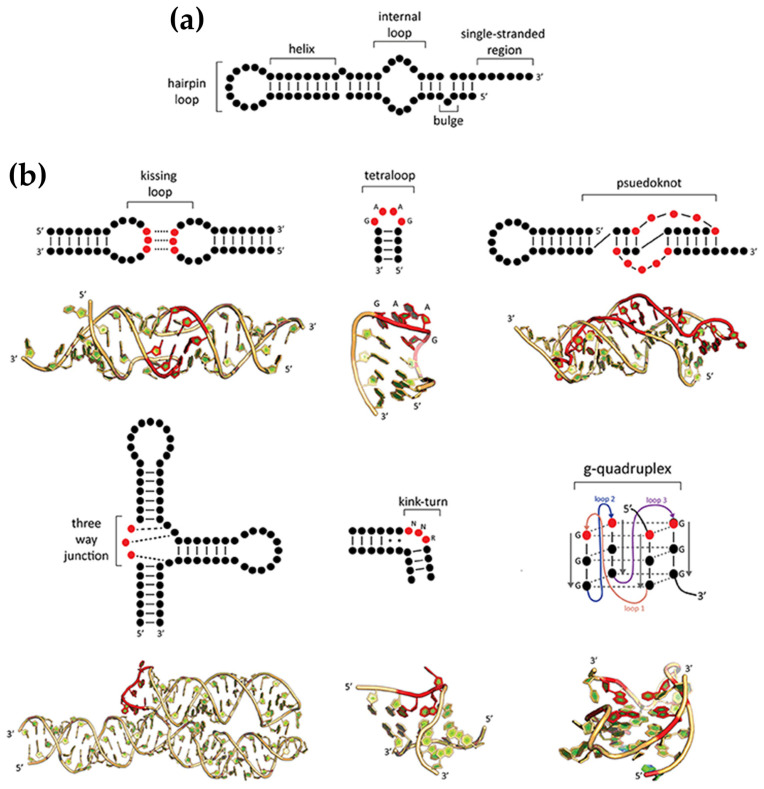
(**a**) Two-dimensional illustrations of common RNA secondary structure motifs. (**b**) Two-dimensional depictions of common RNA secondary and tertiary structure motifs, accompanied by three-dimensional examples from crystal structures. Adapted from [188] under Creative Commons CC BY 4.0 license. Published by Frontiers, 2018.

RNA secondary structures can range from double-stranded helices, loops, junctions, bulges, and single-stranded regions (Figure 36a) [176].

RNA tertiary structures (Figure 36b) are stabilized by various interactions. Coaxial stacking of helices minimizes free energy, imposing overall molecular shapes, such as in tRNA [177,178]. Multi-helix junctions, including three-way and four-way types, constrain topology and facilitate stable tertiary interactions [179,180]. Pseudoknots and kissing loops involve long-range Watson–Crick base pairing or loop–loop interactions, contributing to viral replication, translation regulation, and RNA assembly [181,182,183].

Triple helices, such as those in telomerase RNA, are formed by Watson–Crick base-paired duplexes interacting with a third strand via hydrogen bonds. A-minor interactions insert adenines into minor grooves, compacting RNA and stabilizing tertiary structures [184]. Guanine-rich sequences form quadruplexes, stabilized by ions like potassium, with roles in telomere maintenance and gene regulation [185,186].

Metal ions, both monovalent and divalent, neutralize RNA’s negative charge, stabilize tertiary folds, and participate in catalytic mechanisms [187].

RNA secondary and tertiary structures are critical to its function, influencing processes such as protein synthesis, RNA splicing [189], riboswitch activity [190,191], and other essential metabolic processes [192]. Furthermore, RNA structures provide specific binding platforms for RNA-binding proteins (RBPs), influencing RNA stability, localization, and turnover. This specificity is essential for processes like transcriptional and post-transcriptional regulation [193].

The intricate structural diversity of RNA not only supports its biological functions, but also provides unique opportunities for therapeutic intervention through small molecule interactions. Small molecules can selectively target RNA structures such as internal loops, bulges, riboswitches, and pseudoknots to modulate RNA activity. However, challenges persist due to RNA’s transient structural states and the limited availability of high-resolution RNA–ligand complex data [194]. Computational strategies, including molecular docking, quantitative structure–activity relationship (QSAR) modeling, and ensemble-based virtual screening, have emerged as critical tools to predict binding modes and optimize lead compounds [195,196].

#### Chiroptical and Structural Insights into Porphyrin Interactions with RNA Structures

Given the structural similarities between RNA and DNA, including aromatic nucleobases and a negatively charged phosphate backbone, porphyrins can interact with RNA structures in a manner analogous to their interactions with DNA. However, the vastly different and more variable tertiary structure of RNA introduces additional binding possibilities that are not observed in DNA [197]. Despite these structural distinctions, the fundamental assumptions regarding the ICD signals (Figure 3) remain consistent for porphyrin–RNA interactions, allowing for similar spectroscopic interpretations of binding modes [66,198].

Regarding the interactions between porphyrins and RNA, pioneering works were conducted by several researchers exploring distinct porphyrins, RNA types, and binding modes. In 1988, Foster et al. [197] studied the interaction of H_2_TMPyP4 porphyrin and its Cu(II), Mn(III), and Zn(II) derivatives with yeast tRNA, revealing that these porphyrins bind at a single tertiary structural site near crucial hydrogen bonds, likely in the vicinity of the P10 loop. This binding mode was distinct from classical intercalation or electrostatic interactions, with induced CD signals confirming structural specificity.

Subsequent investigations by Bustamante et al. [198] analyzed water-soluble H_2_TMPyP4, CuTMPyP4, and ZnTMPyP4 interactions with single-stranded poly(A) and poly(C) as well as double-stranded polynucleotides. These studies demonstrated bathochromic shifts, hypochromicity, and pseudo-intercalative binding to purine-rich regions, with Cu derivatives exhibiting prominent induced CD bands. Importantly, both H_2_TMPyP4 and its Zn derivative were found to discriminate between poly(A) and poly(dA), a behavior attributed to the different axial rise per residue of the two polymers. Additionally, the Zn derivative displayed an unprecedented induced negative CD signal, qualifying ZnTMPyP4 as a specific spectroscopic probe for single-stranded poly(A).

In 1996, Uno et al. [199] focused on the binding of H_2_TMPyP4 to different RNA duplexes and RNA–DNA hybrids. They observed single-step binding with significant hypochromicity (40%) and large bathochromic shifts (15 nm). Notably, induced bisignate CD spectra suggested distinct self-stacking interactions along RNA duplex surfaces, whereas negative ICD showed intercalative binding in hybrid systems. This indicates a pronounced conformational difference between RNA duplexes and DNA–RNA hybrids, allowing H_2_TMPyP4 to selectively recognize and distinguish these structures.

However, over the past two decades, relatively fewer studies, compared to DNA, have investigated the interactions between porphyrins and RNA, particularly those focusing on induced circular dichroism (ICD). This scarcity of research may be attributed to challenges in interpreting clear ICD signals specific to porphyrin–RNA complexes or the inherent complexity of RNA’s diverse and dynamic structures. The relevant studies will be examined here.

**Figure 37:**
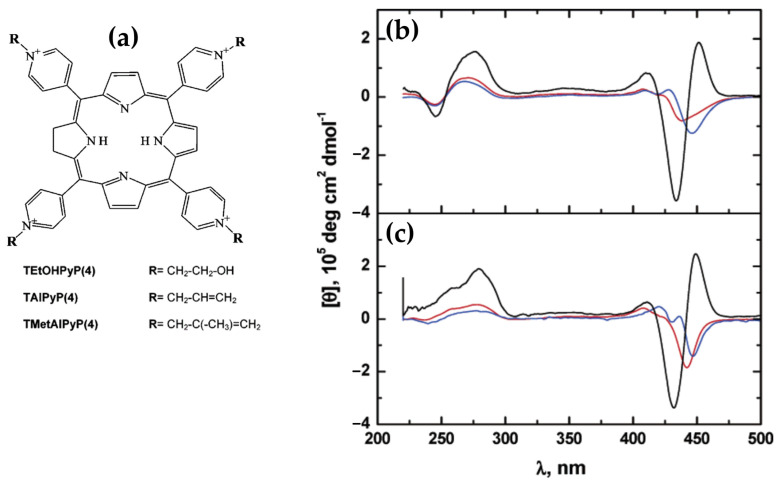
(**a**) Chemical structures of porphyrins TEtOHPyP4, TAlPyP4, and TMetAlPyP4. (**b**) CD spectra of poly(rA)·poly(rU) and (**c**) poly(rI)·poly(rC) complexes saturated with TEtOHPyP4 (red), TAlPyP4 (blue), and TMetAlPyP4 (black). Adapted with permission from ref. [200]. Copyright 2006, American Chemical Society.

In 2006, Ghazaryan et al. [200] investigated the thermodynamics of interactions between water-soluble porphyrins and RNA duplexes, focusing on three derivatives: TEtOHPyP4, TAlPyP4, and TMetAlPyP4 (Figure 37a). Using CD, absorption, and fluorescence spectroscopy, they demonstrated distinct binding modes depending on the porphyrin structure. TEtOHPyP4 and TAlPyP4 intercalated into poly(rA)·poly(rU) and poly(rI)·poly(rC) duplexes, as evidenced by negative ICD signals in the Soret region (Figure 37b,c, red and blue lines), typical of intercalative binding. In contrast, TMetAlPyP4 formed self-stacked aggregates along the RNA helices, producing a strong conservative CD signal with bisignate features (Figure 37b,c, black line).

Thermodynamic analyses revealed that intercalative binding was characterized by favorable binding enthalpies (ΔH), but less favorable entropies (ΔS), while self-stacking interactions showed the opposite trend. These findings highlight the correlation between binding modes and thermodynamic profiles, offering insights into the molecular forces governing porphyrin–RNA interactions. The study underscored the potential of modifying peripheral groups to tailor porphyrin–RNA binding properties, advancing the design of porphyrin-based probes.

**Figure 38:**
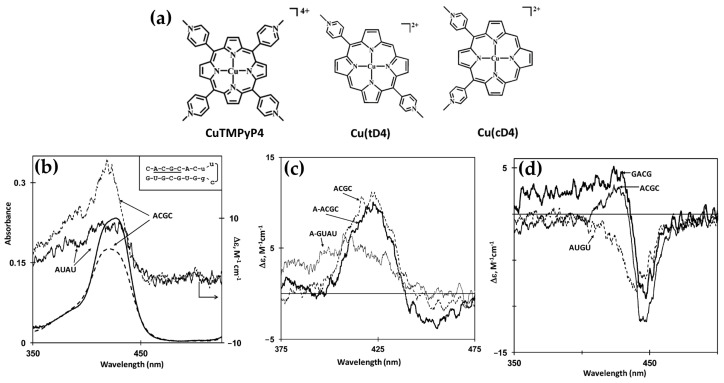
(**a**) Chemical structures of CuTMPyP4, Cu(tD4), and Cu(cD4). (**b**) Limiting absorption spectra of 2.3 μM Cu(tD4) in buffer at q = 46, with RNA hosts AUAU (thick solid line) and ACGC (dashed line); corresponding CD spectra are shown above. (**c**) Induced CD spectra of Cu(cD4) bound to RNA hairpins ACGC (dashed line), A-ACGC (thick solid line), and A-GUAU (thin solid line) at q = 39. (**d**) Induced CD spectra of CuTMPyP4 with G≡C-rich RNA hosts GACG (thick solid line) and ACGC (thin solid line) at q = 23, and with A=U-rich host AUGU (dashed line) at q = 31; all are limiting spectra, where q is the RNA base pair-to-porphyrin concentration ratio. Adapted with permission from ref. [201]. Copyright 2012, American Chemical Society.

In 2012, Briggs et al. [201] explored the binding interactions between cationic copper(II) porphyrins, derived from CuTMPyP4, and two sterically friendlier forms, [*trans*-5,15-di(N-methylpyridinium-4-yl)-porphyrinato]copper(II), Cu(tD4), and [*cis*-5,10-di(N-methylpyridinium-4-yl)porphyrinato]copper(II), Cu(cD4) (Figure 38a), bind to RNA hairpins featuring A-form duplex domains. Using different spectroscopic techniques, they demonstrated that these porphyrins predominantly intercalate into the stem regions of RNA duplexes, irrespective of base pair composition. The induced CD signals were highly dependent on the porphyrin structure and the RNA host’s base content, with positive signals observed for intercalation of Cu(tD4) and Cu(cD4) into RNA (Figure 38b,c), contrasting with the negative signals typical for DNA hosts.

The study highlighted notable differences in the induced CD spectra among the porphyrins. For CuTMPyP4, a bisignate CD signal is induced by G≡C-rich RNA, while A=U-rich RNA produces a strictly negative signal (Figure 38d). These differences are likely due to the low-symmetry environment at the intercalation site, which causes differential responses in the excited state. Additionally, induced CD spectra may reflect local structural variations, such as helix unwinding or base extrusion, required to accommodate sterically demanding ligands. Hypochromic shifts and splitting of the Soret band further confirmed the intercalative binding mode. Additionally, fluorescence emission studies revealed that the copper porphyrins were effectively shielded from solvent quenching, further supporting internalization into the RNA duplexes.

In summary, the study highlights that A-form RNA is highly efficient at internalizing cationic copper(II) porphyrins, often surpassing the binding capabilities of B-form DNA. The unique optical and structural properties observed with copper(II) porphyrins emphasize the complexity of RNA-porphyrin interactions and underscore the need for further research to fully elucidate the mechanisms driving these interactions and interpretate induced CD signals.

**Figure 39:**
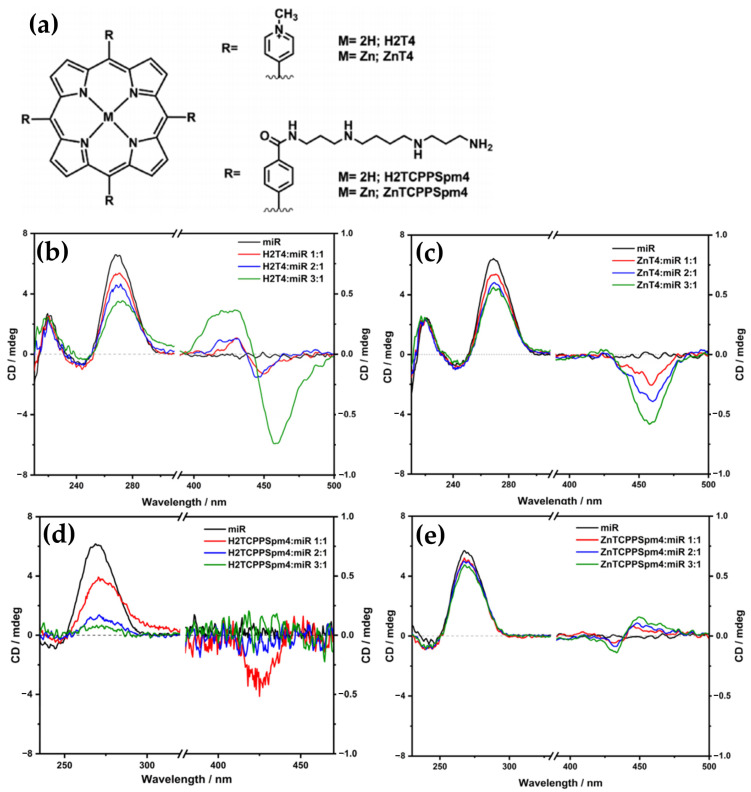
(**a**) Chemical structures of H_2_TMPyP4, ZnTMPyP4, H_2_TCPPSpm4, and ZnTCPPSpm4. (**b**–**e**) CD spectra of miR-26b-5p [2.5 µM] (black curve) in 10 mM PBS (pH = 7.4; [KCl] = 2.7 mM; [NaCl] = 137 mM) with increasing amounts of porphyrins: (**b**) H_2_TMPyP4, (**c**) ZnTMPyP4, (**d**) H_2_TCPPSpm4, and (**e**) ZnTCPPSpm4 at concentrations of 2.5 µM (red curve), 5 µM (blue curve), and 7.5 µM (green curve). Adapted from ref. [202]. under Creative Commons CC BY-NC 3.0 license. Published by Royal Society of Chemistry, 2024.

In 2024, Travagliante et al. [202] investigated the interactions between four achiral porphyrins H_2_TMPyP4, ZnTMPyP4, H_2_TCPPSpm4, and ZnTCPPSpm4 (Figure 39a), and a mature human microRNA (miRNA or miR) sequence. miRNAs are small non-coding RNAs that regulate gene expression by binding to target mRNAs, leading to their degradation or translational repression. Despite their small size (18–24 nucleotides) recent findings suggest that they can adopt highly ordered structures similar to aptamers, enabling diverse functions beyond mRNA recognition. The study employed multiple spectroscopic techniques, including CD, UV-vis, fluorescence, and resonance light scattering (RLS) to elucidate binding mechanisms and thermodynamic effects.

CD experiments revealed that H_2_TMPyP4 binding caused significant alterations in the miRNA secondary structure as evidenced by the decrease of the intense positive band around 270 nm (Figure 39b). Weak bisignate ICD signals at lower porphyrin-to-miRNA ratios (1:1, 2:1) suggested initial porphyrin dimer formation with miRNA. At higher ratios (≥3:1), a stronger bisignate ICD signal emerged (Figure 39b), consistent with porphyrin stacking onto the miRNA structure, driven by electrostatic interactions.

ZnTMPyP4, constrained by its pentacoordinated zinc center, exhibited a weaker interaction mode. Its weak and negative ICD signals (Figure 39c) suggested pseudo-intercalation near unpaired regions at the 5′ and 3′ ends of the miRNA. This binding mode was less disruptive to the miRNA structure, as evidenced by weakly perturbed CD band around 270 nm.

H_2_TCPPSpm4 demonstrated a unique ability to destabilize miRNA secondary structures. CD signals showed a strong reduction in the band at 270 nm (Figure 39d), reflecting disrupted base pairing and the transition to a more random coil conformation. A weak negative ICD signal in the Soret region (Figure 39d) suggested intercalation or stacking interactions, but this disappeared at higher porphyrin-to-miRNA ratios, indicating external binding predominated as the spermine groups disrupted hydrogen bonding.

ZnTCPPSpm4 formed aggregates on the miRNA structure, producing a weak bisignate ICD signal in the Soret band (Figure 39e), without perturbating the miRNA secondary structure. This indicated an organized aggregate orientation on the miRNA backbone, distinct from the aggregation behavior of H_2_T4.

These findings highlighted the versatility of porphyrins as chiroptical probes, with their ICD responses reflecting distinct binding modes ranging from intercalation and pseudo-intercalation to aggregation. The study emphasized the structural adaptability of miRNAs in accommodating porphyrins and their potential for use in RNA-targeted applications.

**Figure 40:**
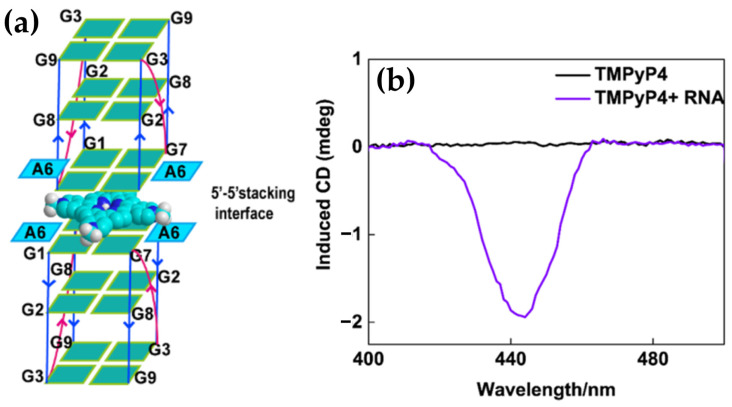
(**a**) Illustration of H_2_TMPyP4 intercalating at 5′-5′ stacking interface of TERRA G-quadruplex dimer. (**b**) Induced circular dichroism (ICD) spectra of 4 μM H_2_TMPyP4 in absence and presence of 16 μM TERRA G-quadruplex dimer. Adapted with permission from ref. [203]. Copyright 2019, American Chemical Society.

**Figure 41:**
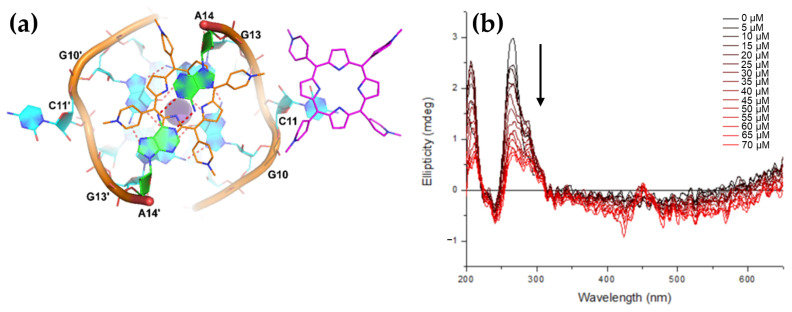
(**a**) Crystal structure of the RNA- H_2_TMPyP4 complex (PDB ID: 6JJH), showing two distinct binding modes of H_2_TMPyP4. The H_2_TMPyP4 molecule in orange is intercalated between A14, A14′, and guanine bases (G10, G10′, G13, and G13′) of the first G-tetrad, while the H_2_TMPyP4 molecule in magenta is π–π stacked with a C11 base. (**b**) CD titration of PQS18-1 RNA (10 μM) with H_2_TMPyP4 concentrations ranging from 0 to 70 μM, The arrow indicates the change in the CD signal with increasing porphyrin concentration. Adapted with permission from ref. [204]. Copyright 2022, American Chemical Society.

The last two papers of this section explored the interactions between H_2_TMPyP4 and two distinct RNA G4 structures. The first one is a dimeric RNA G4 formed by a 10-nt sequence, r(GGGUUAGGGU), derived from Telomeric Repeat-containing RNA (TERRA) (Figure 40a), which consists of long non-coding RNAs transcribed from telomeres [203]. The second one is the RNA G4-forming sequence PQS18-1 (r(GGCUCGGCGGCGGA); PDB ID: 6JJH, 6JJI) (Figure 41a), a bimolecular, all-parallel-stranded G4-RNA consisting of four stacked tetrads with three K+ ions aligned along its central axis [204].

The first study [203] revealed that H_2_TMPyP4 preferentially intercalates into the 5′-5′ stacking interface of the TERRA G-quadruplex dimer (Figure 40a). This interaction is driven by the enhanced π–π stacking provided by the A·(G·G·G·G)·A hexads, as confirmed by negative ICD signal in the Soret region (Figure 40b), hypochromicity, and redshifts in the UV-Vis absorption spectrum. NMR data further validated the specificity of this binding site, highlighting H_2_TMPyP4′s potential for targeting higher-order RNA G-quadruplex structures.

In contrast, the second study [204] focused on the PQS18-1 RNA G4 and demonstrated that H_2_TMPyP4 binding leads to structural remodeling of the G4. CD spectra showed a decrease in ellipticity at 264 nm, accompanied by the emergence of a shoulder at 295 nm (Figure 41b), indicative of a transition from a parallel to an antiparallel conformation. This interaction, characterized by weak ICD signals and exciton splitting, suggested H_2_TMPyP4 forms dimers or aggregates within the G4 grooves or along the external stacking sites, supported by molecular dynamics and crystal structure studies.

Together, these findings underscore the diversity in binding modes and structural effects of H_2_TMPyP4 on RNA G-quadruplexes. While the TERRA dimer provides a well-defined intercalative binding site, the PQS18-1 G4 highlights the dynamic interplay between ligand binding and RNA structural transitions. These insights pave the way for further exploration of porphyrins as versatile tools for studying and targeting RNA G-quadruplexes in biological and therapeutic contexts.

## 3. Conclusions

In conclusion, this review summarized the versatility and capability of porphyrins as chiroptical probes for DNA and RNA, offering a comprehensive understanding of their non-covalent interactions with nucleic acids. Porphyrins are ideal compounds for these studies due to their unique photophysical properties, synthetic adaptability, and remarkable ability to interact with diverse nucleic acid conformations, including B-, Z-, and A-DNA, G-quadruplexes, i-motifs, and RNA structures. Their ability to acquire induced circular dichroism (ICD) signals in the Soret region is a powerful tool to obtain more information regarding nucleic acid structures and binding mechanisms. Although the past decade has seen significant advancements in porphyrin-nucleic acid interaction studies, there remains a notable limitation in the exploration of porphyrins as chiroptical probes for certain nucleic acid structures, particularly A-DNA, i-motif DNA, and RNA. This gap underscores the need for further research to fully understand their binding behaviors and to harness their potential as probes for these less-studied structures. Expanding the range of porphyrin derivatives and fine-tuning their molecular design could enhance their selectivity and sensitivity in probing complex nucleic acid architectures.

Prospectively, porphyrins hold significant promise for applications beyond structural studies. One of the most exciting prospects is their integration into diagnostic platforms for detecting specific nucleic acid conformations associated with diseases [205]. Additionally, the ability of porphyrins to generate singlet oxygen upon light activation makes them excellent candidates for photodynamic therapy (PDT), particularly when selectively bound to nucleic acids. Recent studies have demonstrated that porphyrin–DNA and porphyrin–RNA complexes can serve as targeted photosensitizers, enabling site-specific photodamage in cancerous or virally infected cells [206,207,208,209].

Future directions in porphyrin research should explore hybrid systems combining porphyrins with nanomaterials, such as graphene and metal–organic frameworks, to improve their stability and specificity in biological environments [210,211,212]. Additionally, advances in computational modeling and machine learning could facilitate the design of porphyrin derivatives with enhanced chiroptical properties, enabling their use in personalized medicine and targeted diagnostics.

By addressing these challenges and expanding their applications, porphyrin-based chiroptical probes could revolutionize nucleic acid research, diagnostics, and therapeutics, solidifying their role as indispensable tools in biochemistry and medicine.

## Data Availability

No new data were created or analyzed in this study. Data sharing is not applicable to this article.
